# Supplementary material for: Copper-Catalyzed Redox Coupling of Nitroarenes with Sodium Sulfinates
Source: Molecules. 2019 Apr 10;24(7):1407. doi: 10.3390/molecules24071407 (PMC6479299; doi:10.3390/molecules24071407)
Supplement: Supplementary file 1 [file molecules-24-01407-s001.pdf]

Supporting information for

Copper-Catalyzed Redox Coupling of Nitroarenes with Sodium Sulfinates

Saiwen Liu [1,*], Ru Chen [2] and Jin Zhang [1,*]

[1] College of Materials and Chemical Engineering, Hunan City University, Yiyang, 413000, China

[2] Yiyang Agriculture Products Quality Detect Center, Yiyang, Hunan 413000, China

* Correspondence: liusaiwen7@163.com

Table of Contents

Copies of ^{1}H and ^{13}C NMR spectra of products S2-S22

^{1}H and ^{13}C NMR spectra

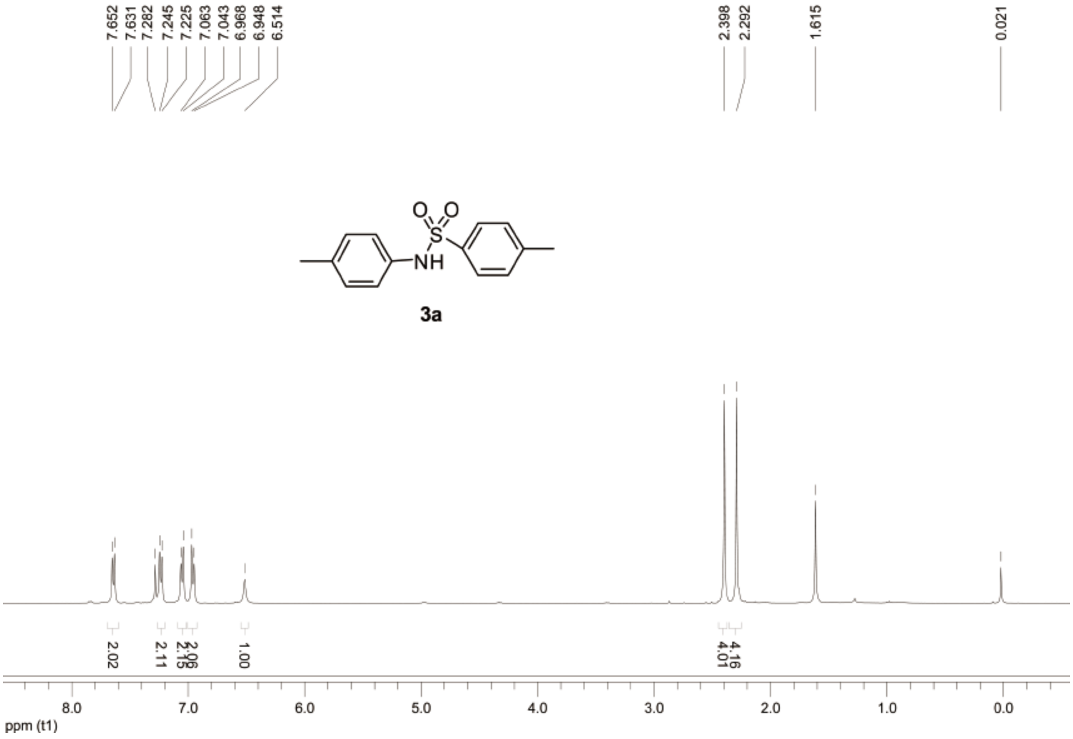

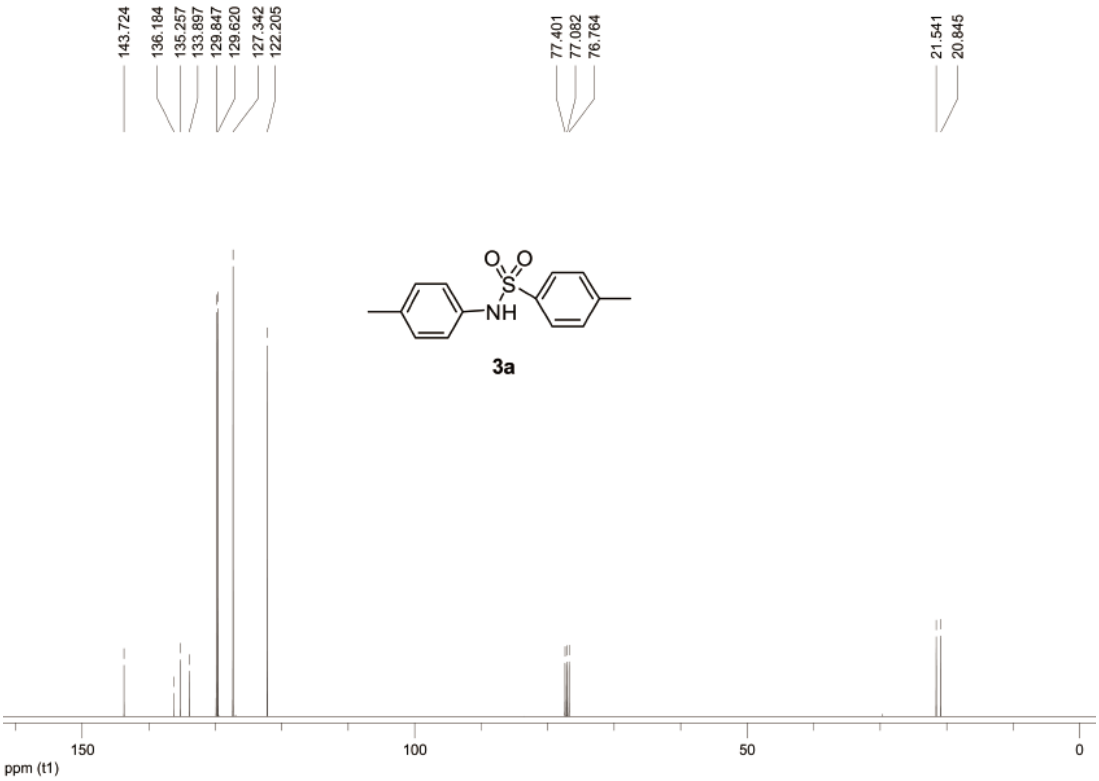

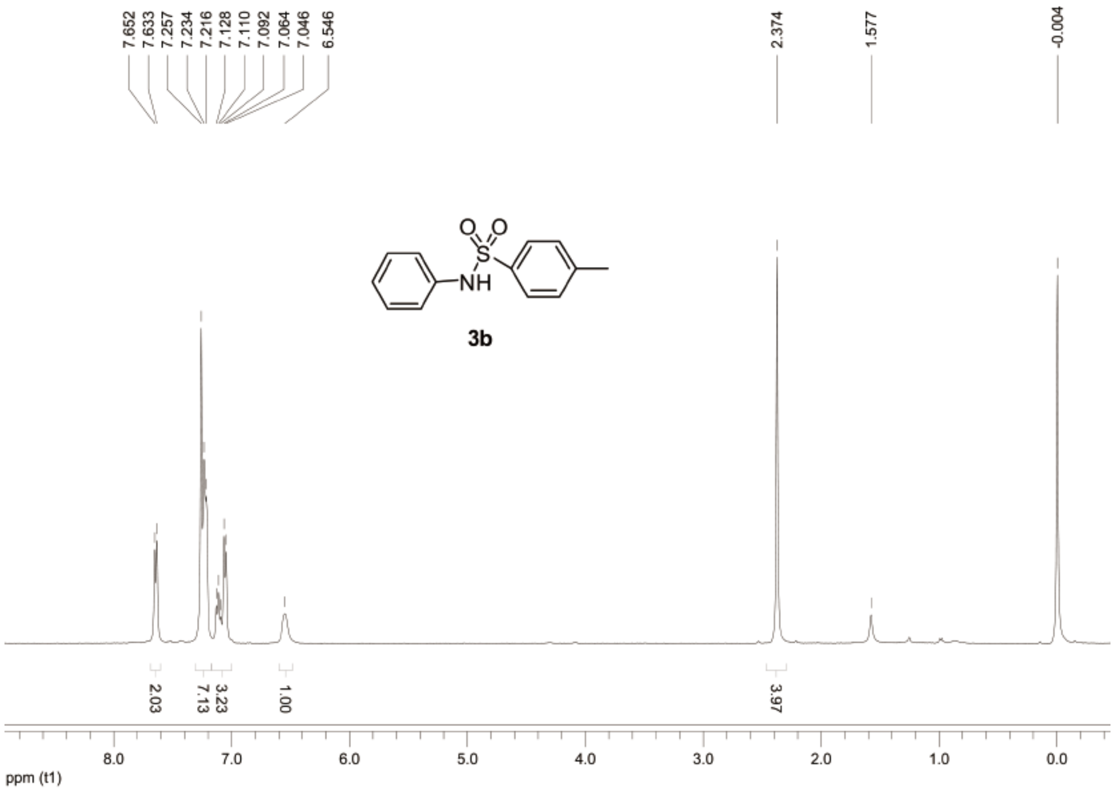
7.652
7.633
7.257
7.234
7.216
7.128
7.110
7.092
7.064
7.046
6.546
2.374
1.577
-0.004
3b
2.03
7.13
3.23
1.00
3.97
8.0
7.0
6.0
5.0
4.0
3.0
2.0
1.0
0.0
ppm (t1)

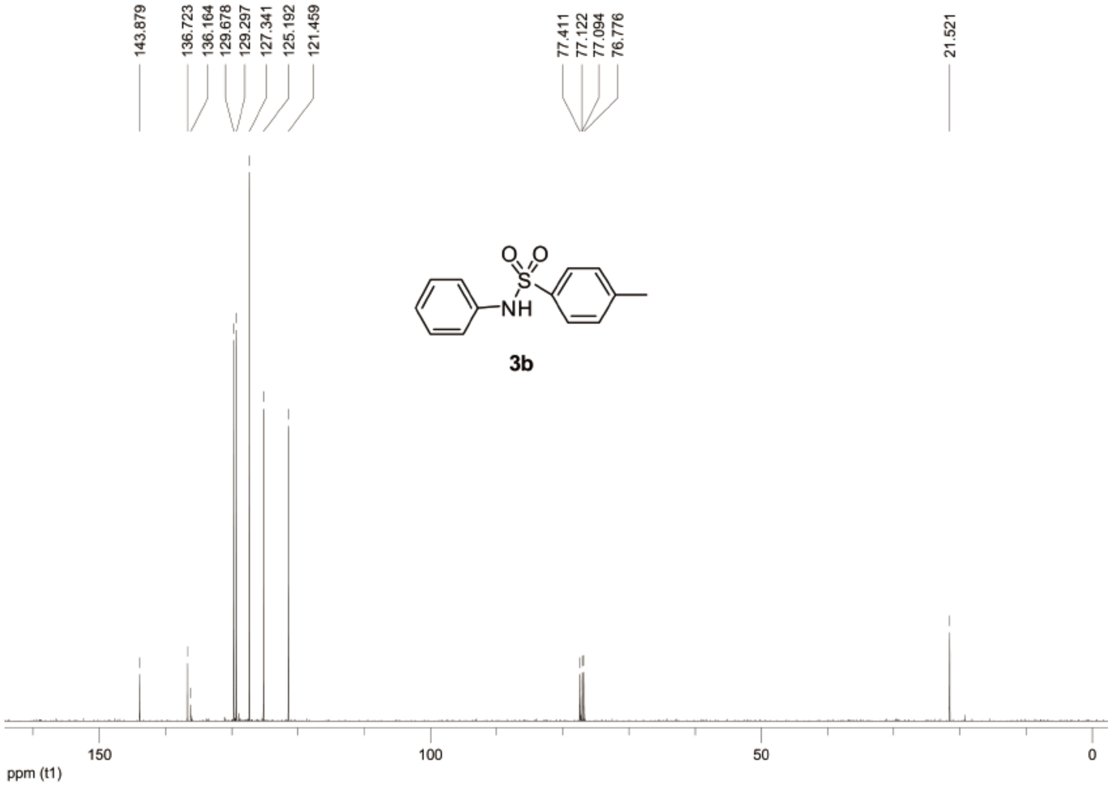
143.879
136.723
136.164
129.678
129.297
127.341
125.192
121.459
77.411
77.122
77.094
76.776
21.521
3b
150
100
50
0
ppm (t1)

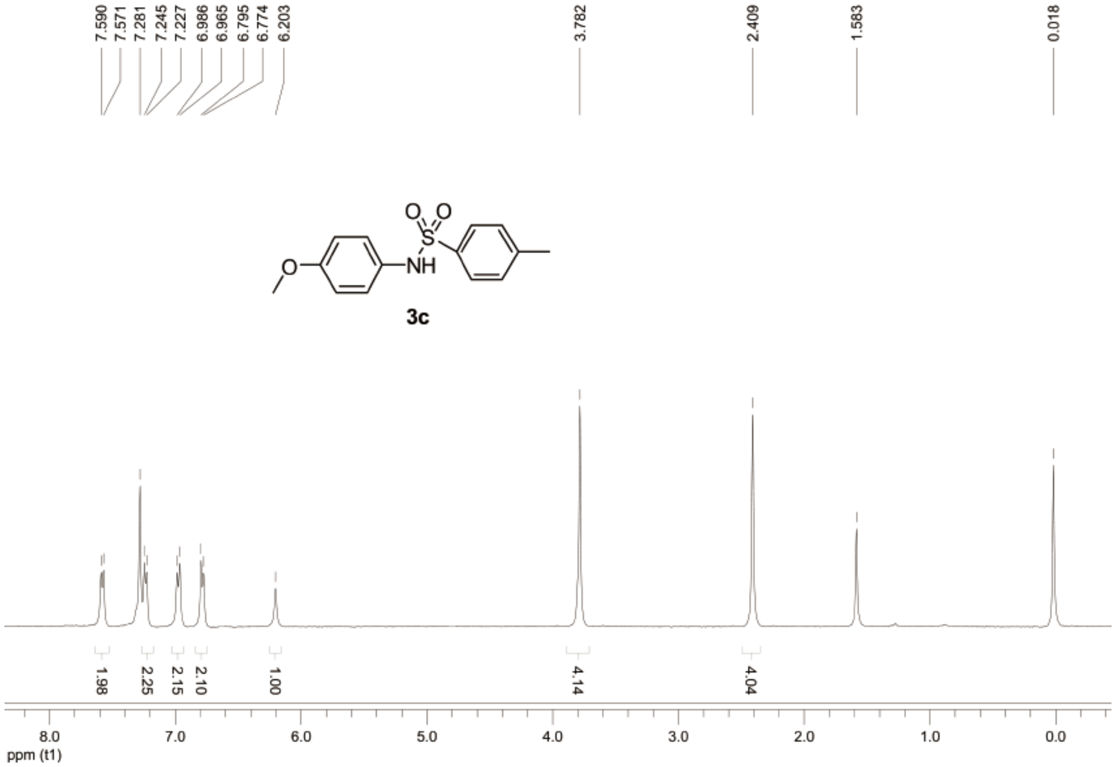

7.590
7.571
7.281
7.245
7.227
6.986
6.965
6.795
6.774
6.203
3.782
2.409
1.583
0.018
O
S
O
NH
O
3c
1.98
2.25
2.15
2.10
1.00
4.14
4.04
8.0
7.0
6.0
5.0
4.0
3.0
2.0
1.0
0.0
ppm (t1)

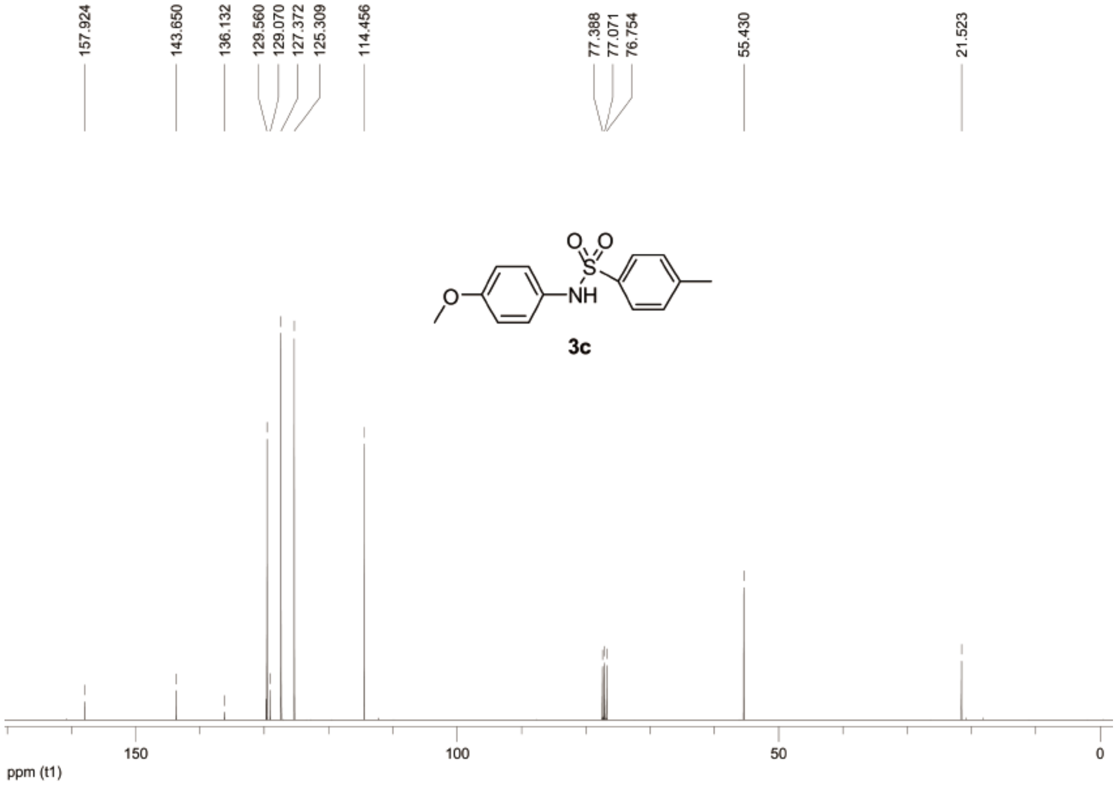

157.924
143.650
136.132
129.560
129.070
127.372
125.309
114.456
77.388
77.071
76.754
55.430
21.523
O
S
O
NH
O
3c
150
100
50
0
ppm (t1)

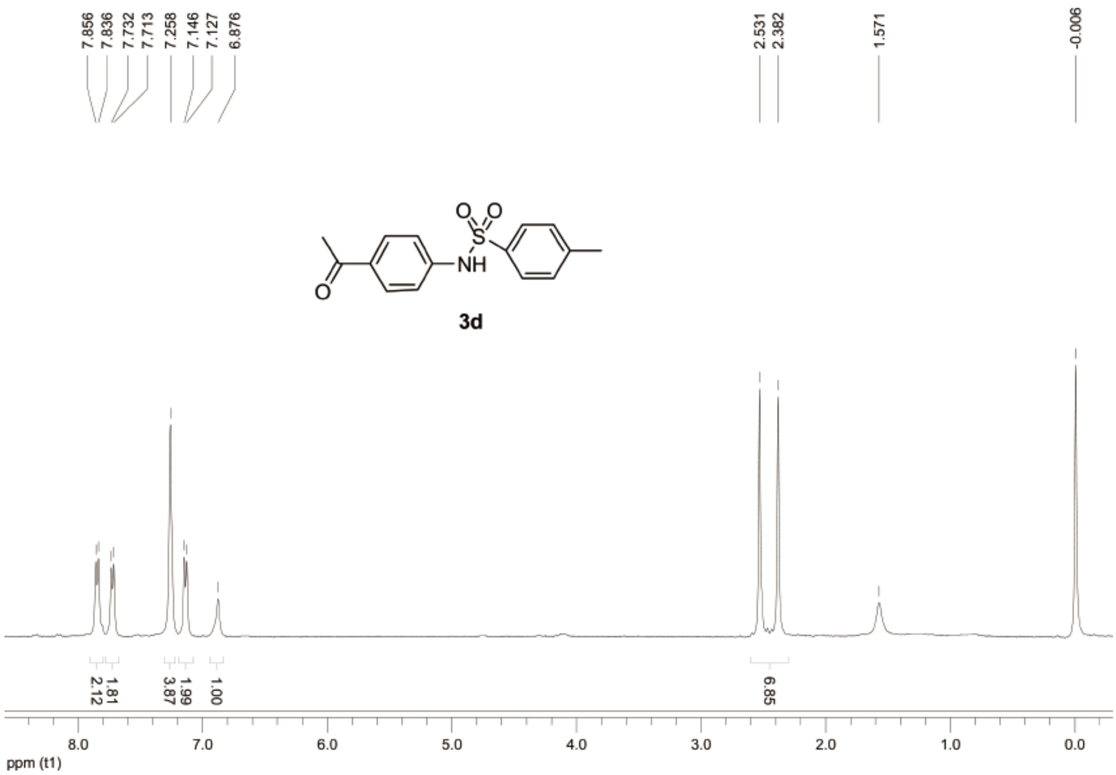
7.856
7.836
7.732
7.713
7.258
7.146
7.127
6.876
2.531
2.382
1.571
-0.006
3d
2.12
1.81
3.87
1.99
1.00
6.85
8.0
7.0
6.0
5.0
4.0
3.0
2.0
1.0
0.0
ppm (t1)

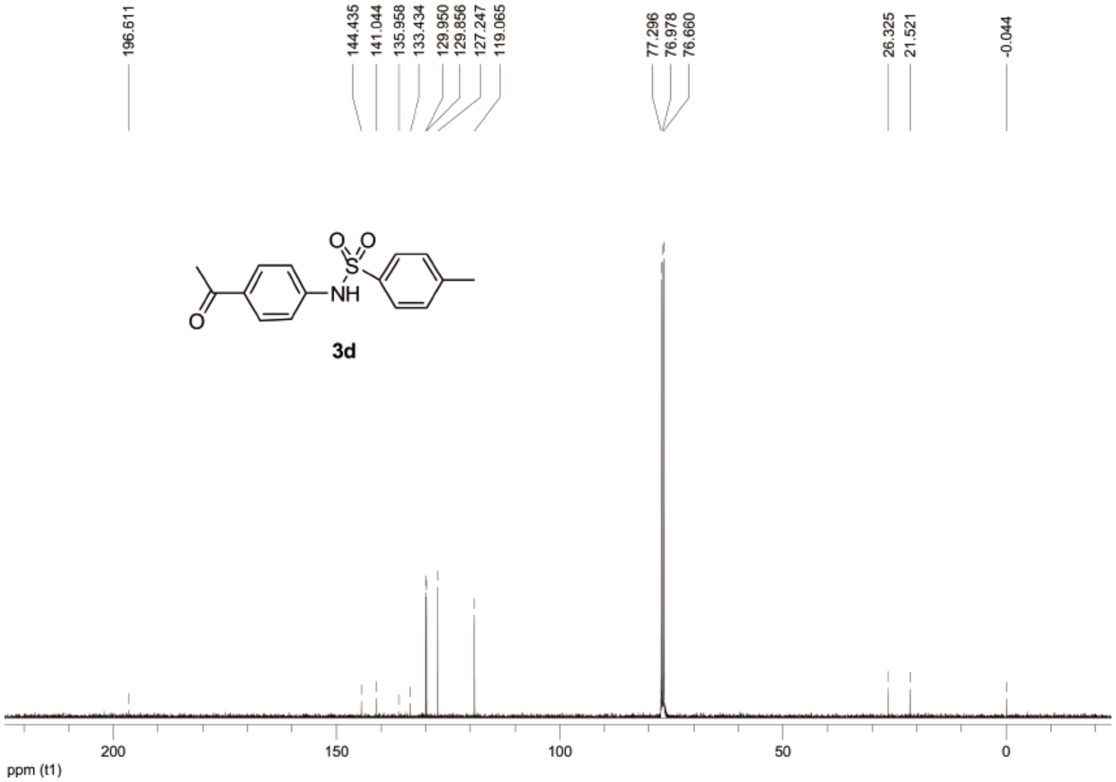
196.611
144.435
141.044
135.958
133.434
129.950
129.856
127.247
119.065
77.296
76.978
76.660
26.325
21.521
-0.044
3d
200
150
100
50
0
ppm (t1)

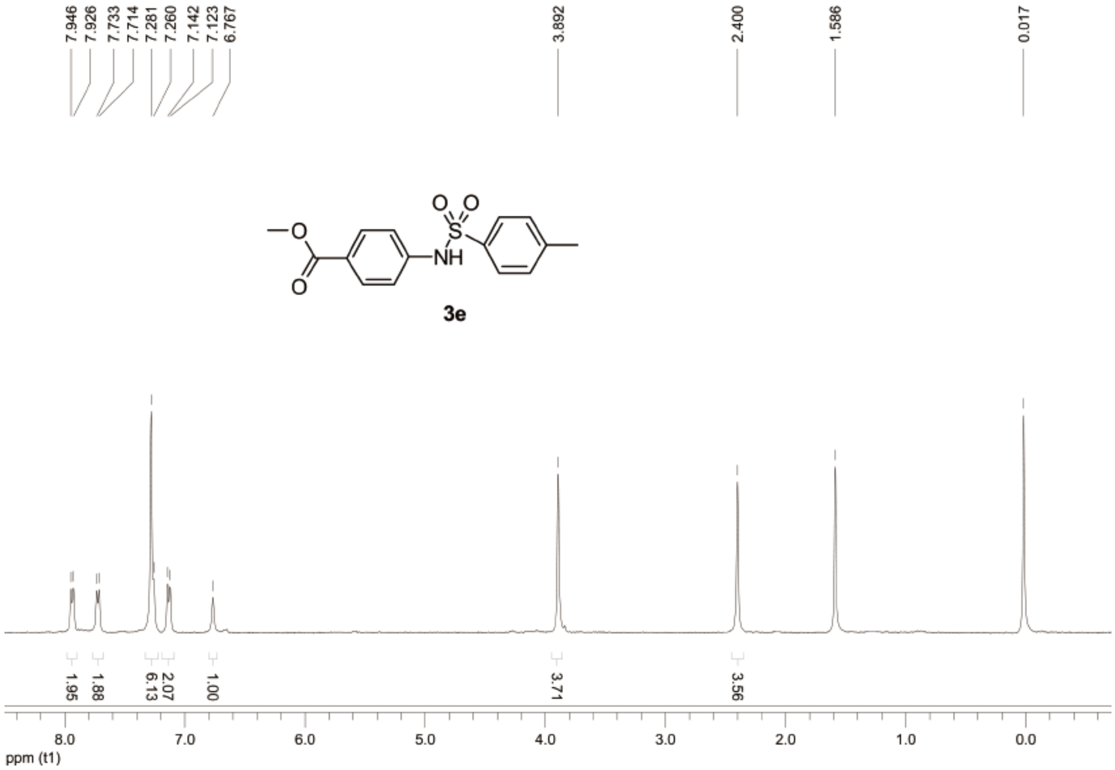
7.946
7.926
7.733
7.714
7.281
7.260
7.142
7.123
6.767
3.892
2.400
1.586
0.017
O
S
O
O
NH
O
3e
1.95
1.88
6.13
2.07
1.00
3.71
3.56
8.0
7.0
6.0
5.0
4.0
3.0
2.0
1.0
0.0
ppm (t1)

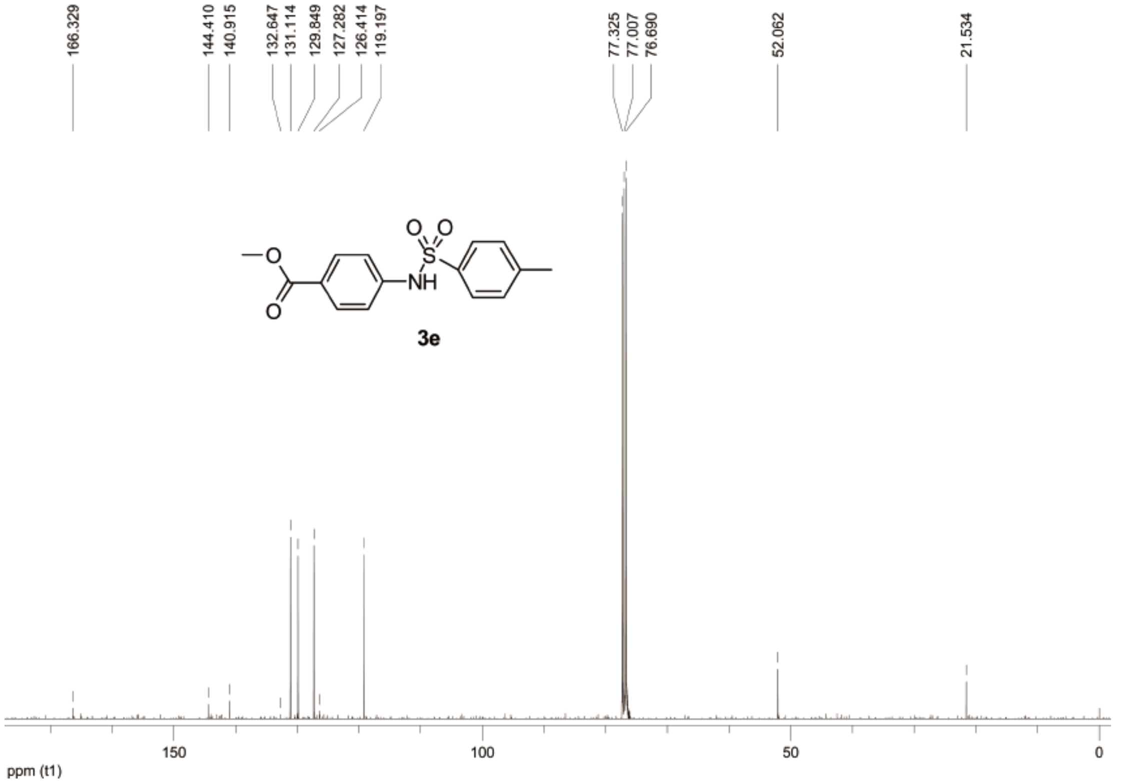
166.329
144.410
140.915
132.647
131.114
129.849
127.282
126.414
119.197
77.325
77.007
76.690
52.062
21.534
O
S
O
O
NH
O
3e
150
100
50
0
ppm (t1)

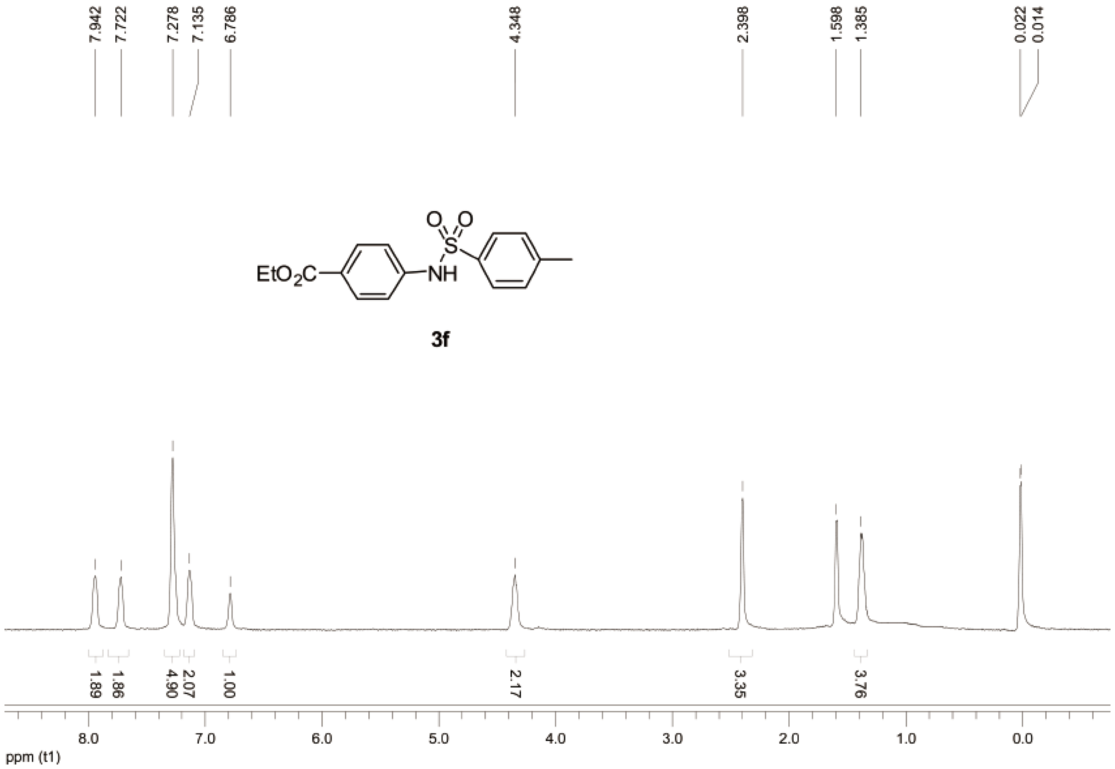
7.942
7.722
7.278
7.135
6.786
4.348
2.398
1.598
1.385
0.022
0.014
EtO2C
O
O
S
NH
3f
1.89
1.86
4.90
2.07
1.00
2.17
3.35
3.76
8.0
7.0
6.0
5.0
4.0
3.0
2.0
1.0
0.0
ppm (t1)

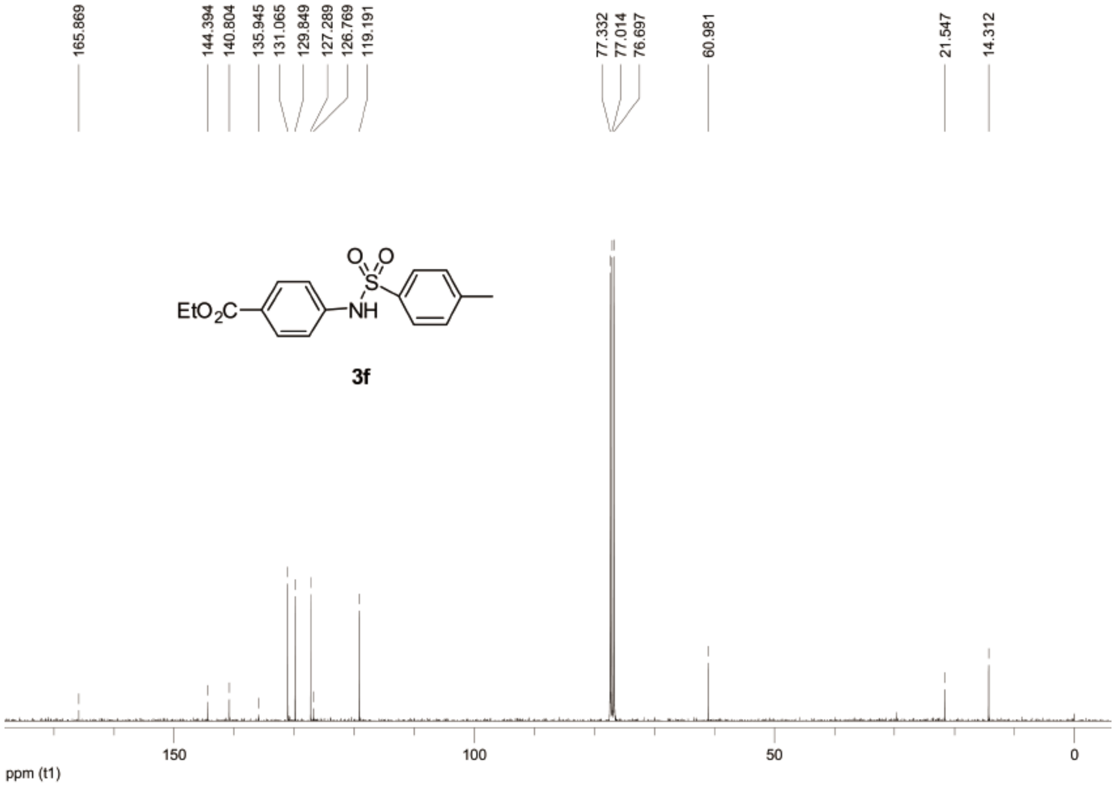
165.869
144.394
140.804
135.945
131.065
129.849
127.289
126.769
119.191
77.332
77.014
76.697
60.981
21.547
14.312
EtO2C
O
O
S
NH
3f
150
100
50
0
ppm (t1)

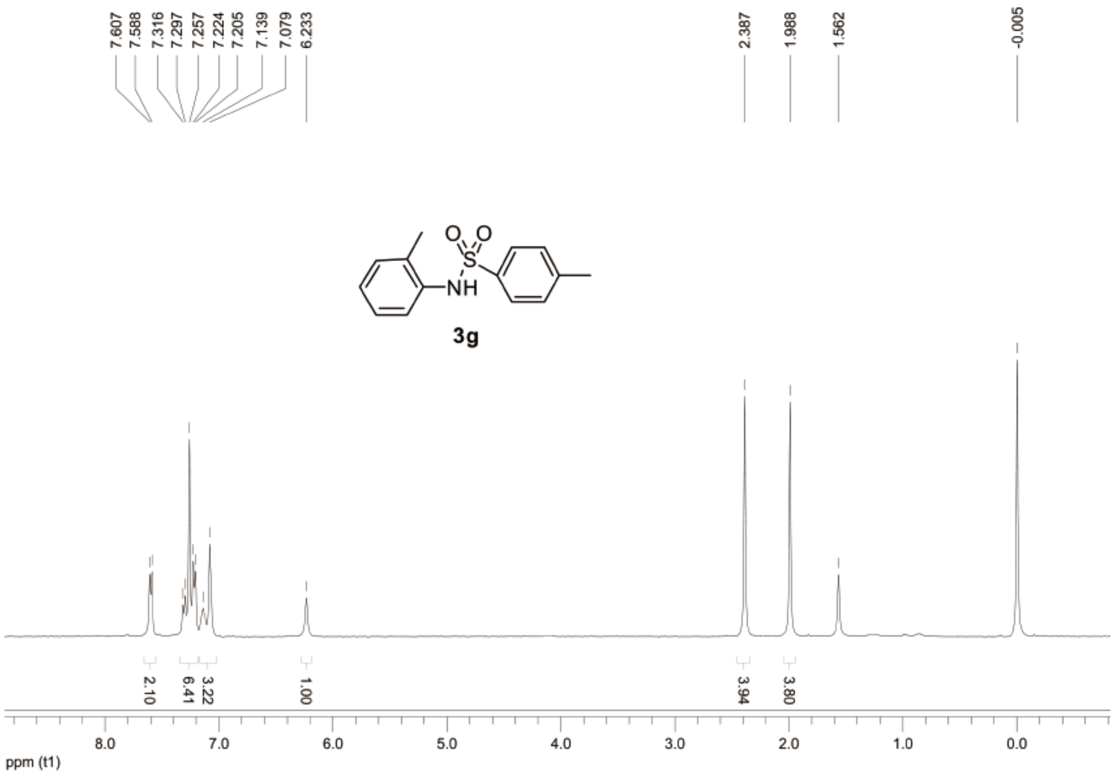
7.607
7.588
7.316
7.297
7.257
7.224
7.205
7.139
7.079
6.233
2.387
1.988
1.562
-0.005
O
O
S
NH
3g
2.10
6.41
3.22
1.00
3.94
3.80
8.0
7.0
6.0
5.0
4.0
3.0
2.0
1.0
0.0
ppm (t1)

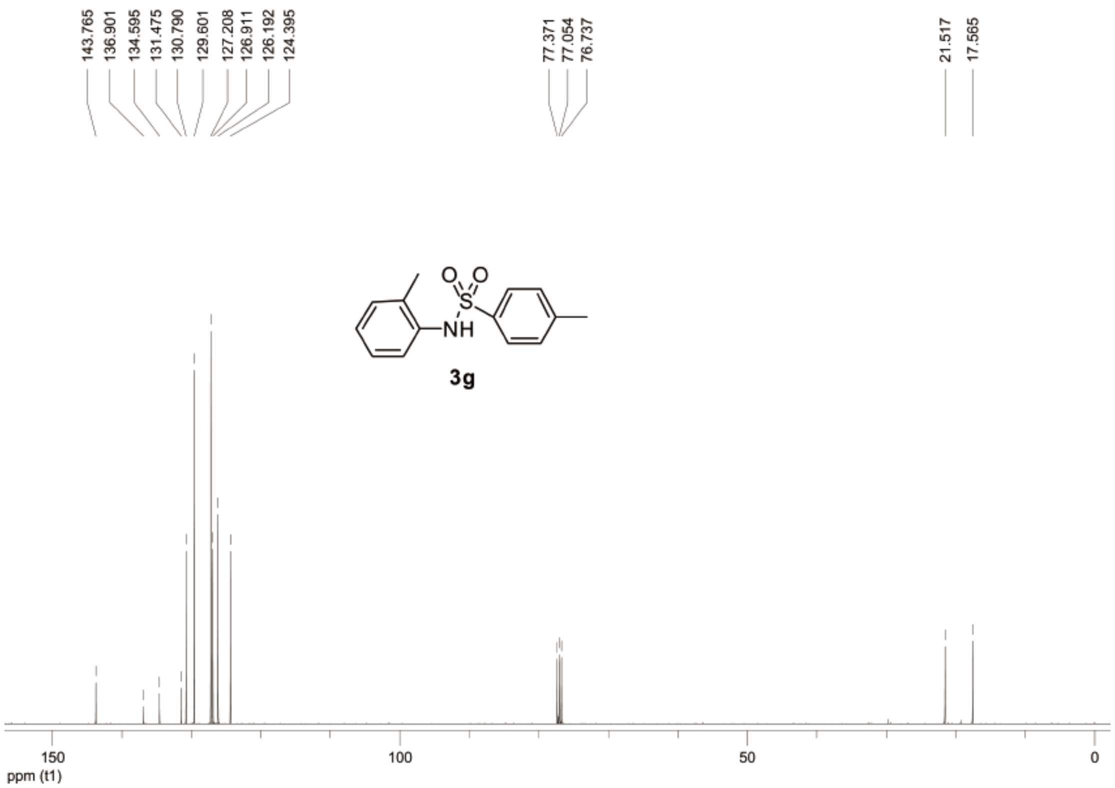
143.765
136.901
134.595
131.475
130.790
129.601
127.208
126.911
126.192
124.395
77.371
77.054
76.737
21.517
17.565
O
O
S
NH
3g
150
100
50
0
ppm (t1)

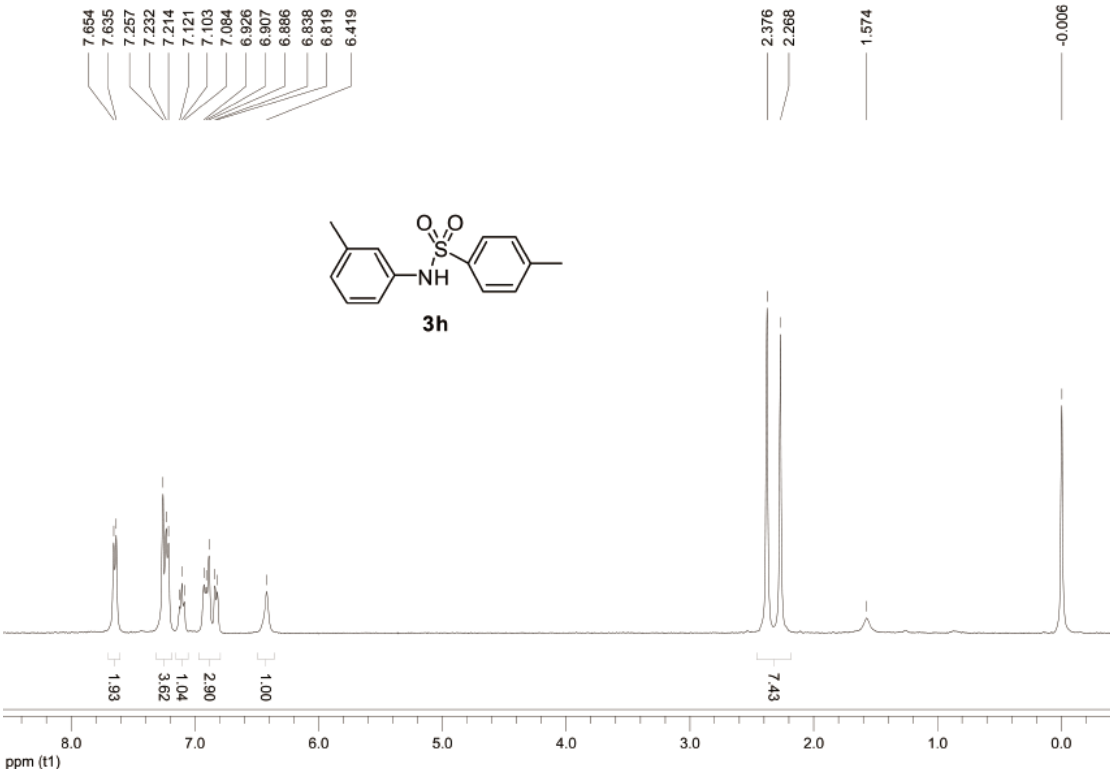
7.654
7.635
7.257
7.232
7.214
7.121
7.103
7.084
6.926
6.907
6.886
6.838
6.819
6.419
2.376
2.268
1.574
-0.006
3h
1.93
3.62
1.04
2.90
1.00
7.43
8.0
7.0
6.0
5.0
4.0
3.0
2.0
1.0
0.0
ppm (t1)

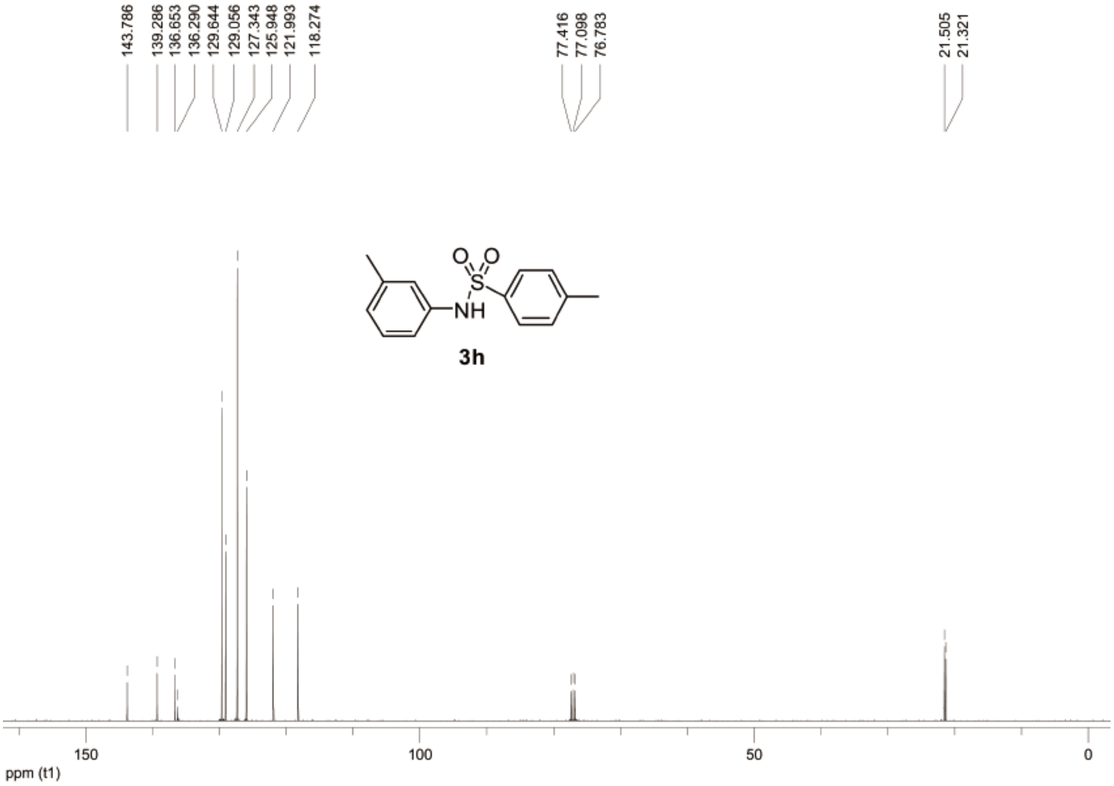
143.786
139.286
136.653
136.290
129.644
129.056
127.343
125.948
121.993
118.274
77.416
77.098
76.783
21.505
21.321
3h
150
100
50
0
ppm (t1)

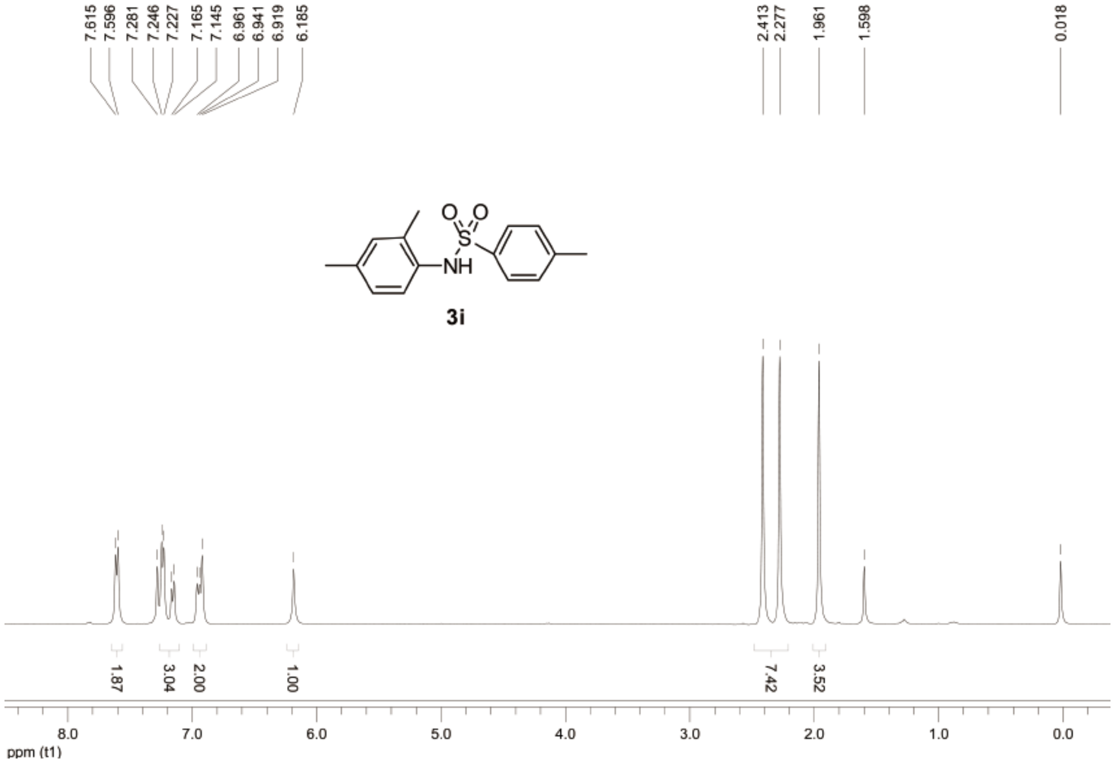
7.615
7.596
7.281
7.246
7.227
7.165
7.145
6.961
6.941
6.919
6.185
2.413
2.277
1.961
1.598
0.018
3i
1.87
3.04
2.00
1.00
7.42
3.52
8.0
7.0
6.0
5.0
4.0
3.0
2.0
1.0
0.0
ppm (t1)

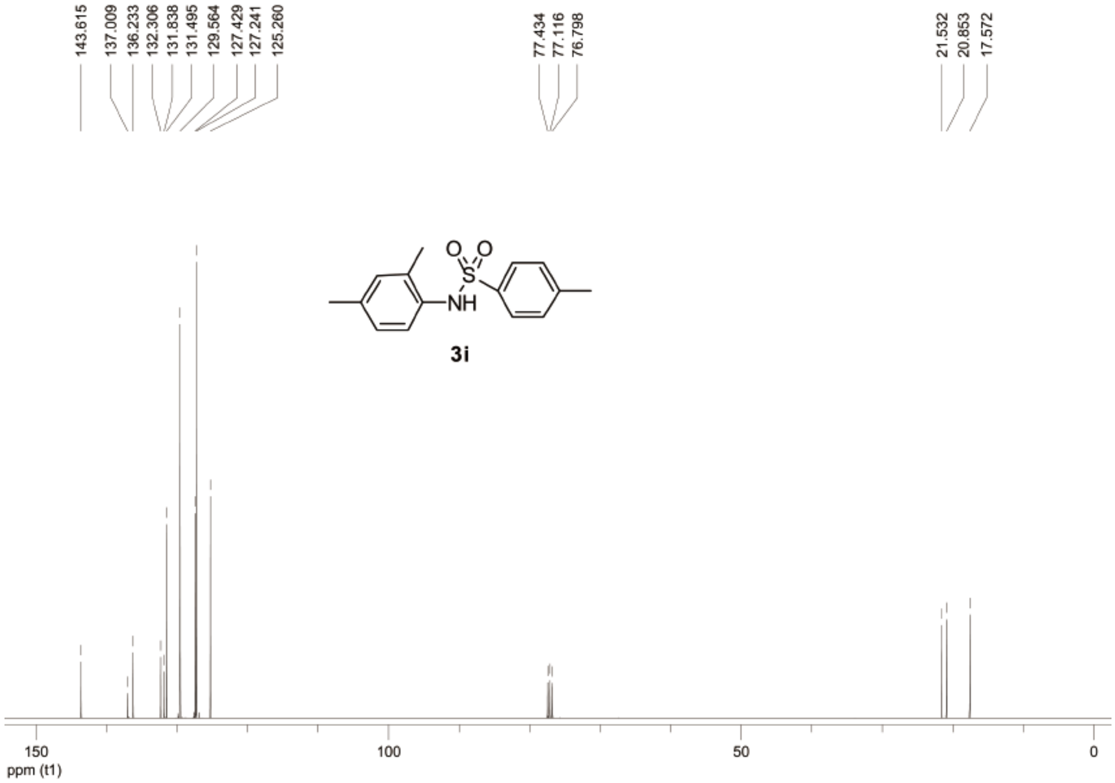
143.615
137.009
136.233
132.306
131.838
131.495
129.564
127.429
127.241
125.260
77.434
77.116
76.798
21.532
20.853
17.572
3i
150
100
50
0
ppm (t1)

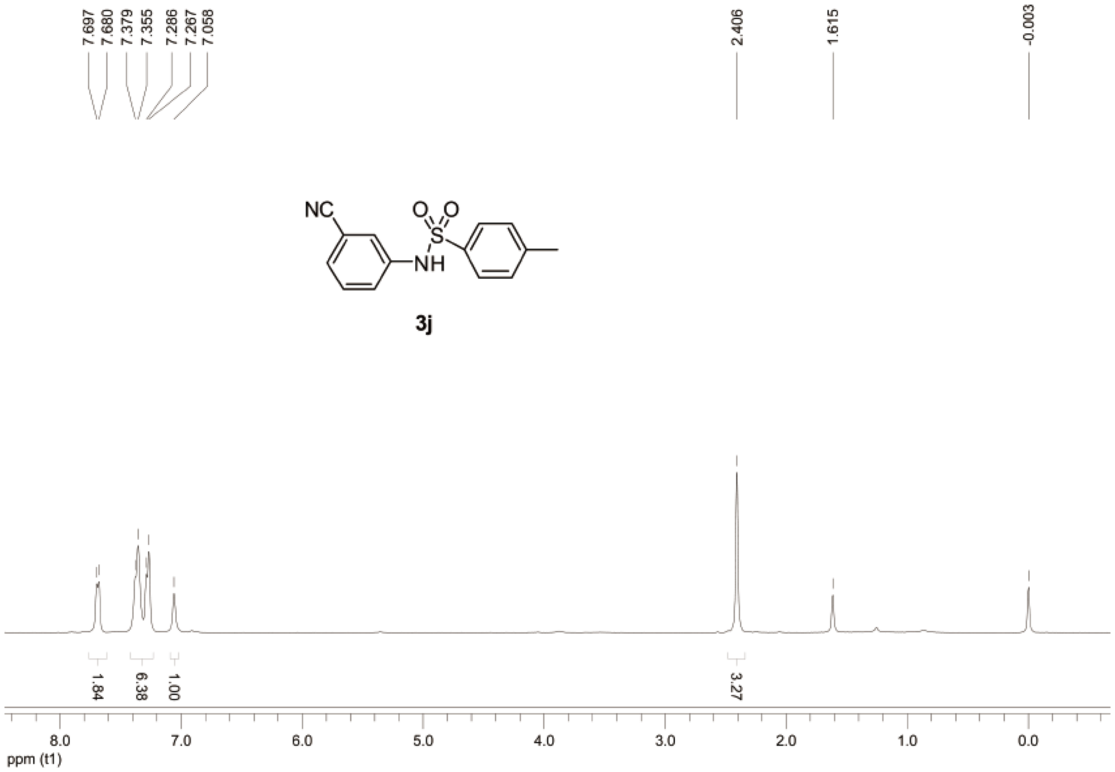
7.697
7.680
7.379
7.355
7.286
7.267
7.058
2.406
1.615
-0.003
NC
O
O
S
NH
3j
1.84
6.38
1.00
3.27
8.0
7.0
6.0
5.0
4.0
3.0
2.0
1.0
0.0
ppm (t1)

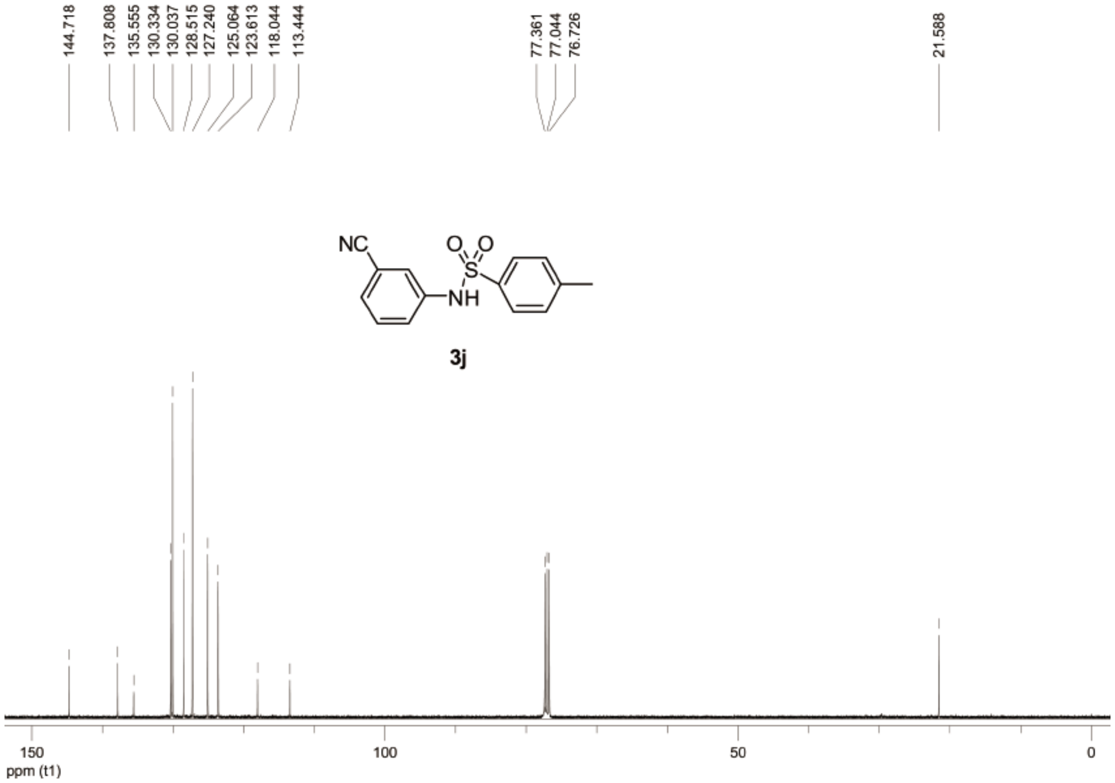
144.718
137.808
135.555
130.334
130.037
128.515
127.240
125.064
123.613
118.044
113.444
77.361
77.044
76.726
21.588
NC
O
O
S
NH
3j
150
100
50
0
ppm (t1)

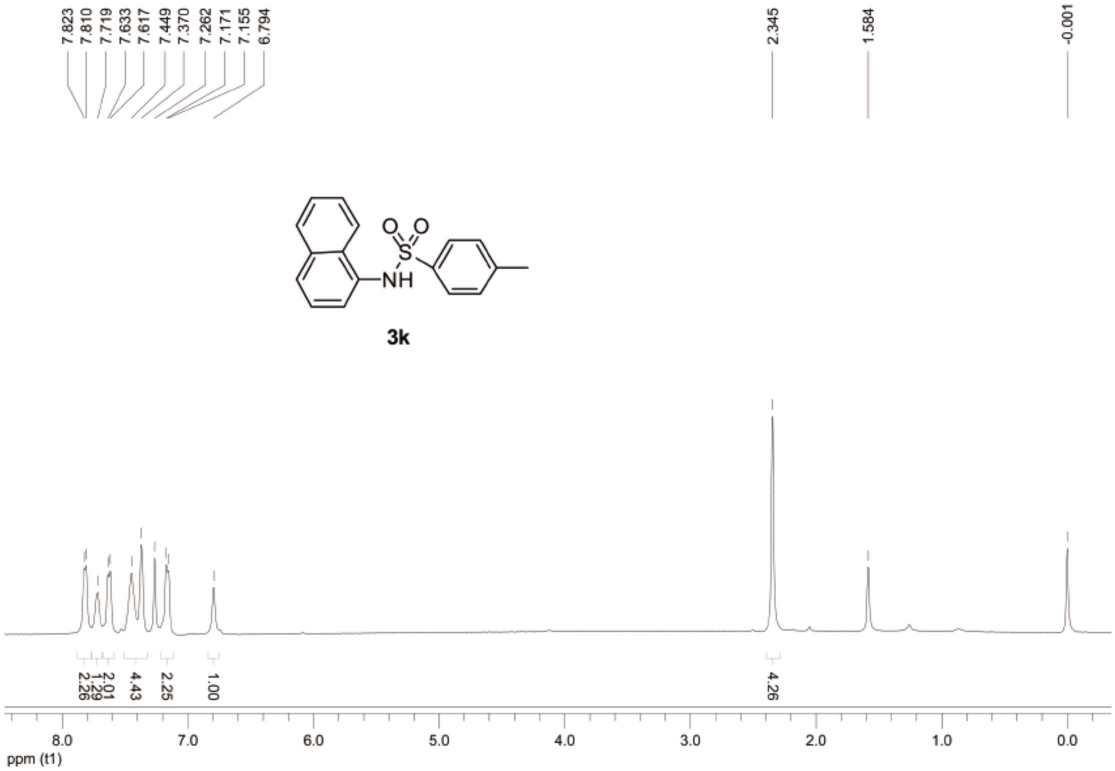
7.823
7.810
7.719
7.633
7.617
7.449
7.370
7.262
7.171
7.155
6.794
2.345
1.584
-0.001
O
O
S
NH
3k
2.26
1.29
2.01
4.43
2.25
1.00
4.26
8.0
7.0
6.0
5.0
4.0
3.0
2.0
1.0
0.0
ppm (t1)

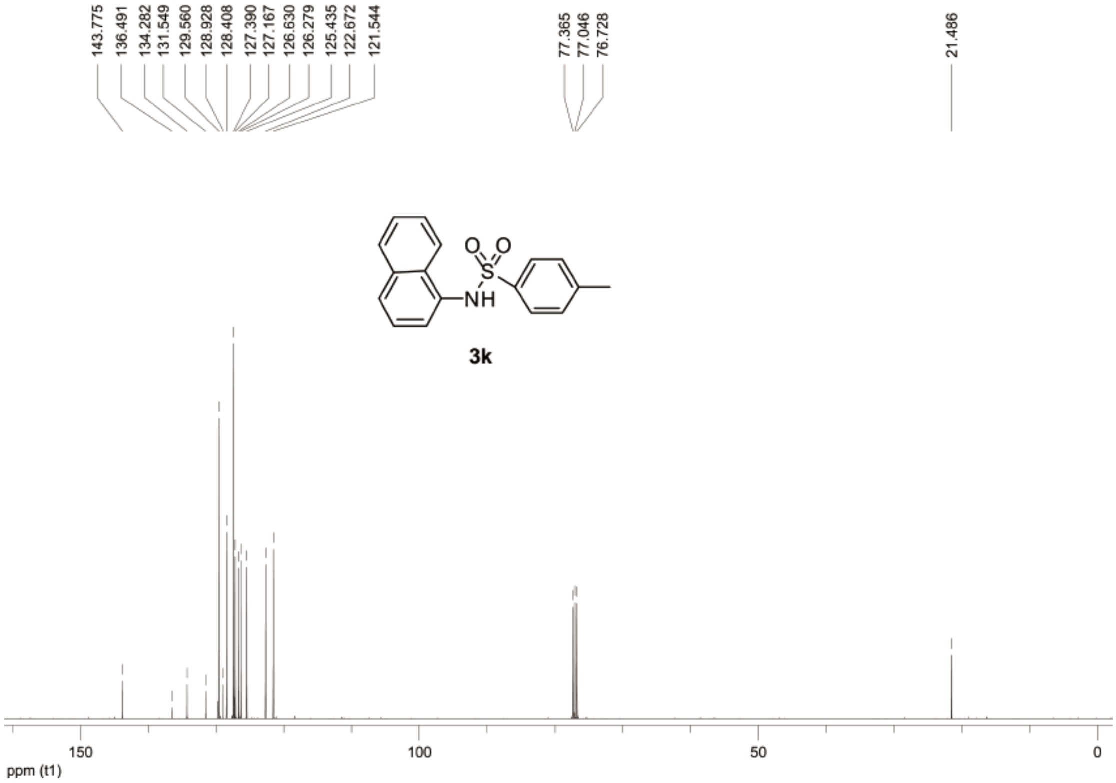
143.775
136.491
134.282
131.549
129.560
128.928
128.408
127.390
127.167
126.630
126.279
125.435
122.672
121.544
77.365
77.046
76.728
21.486
O
O
S
NH
3k
150
100
50
0
ppm (t1)

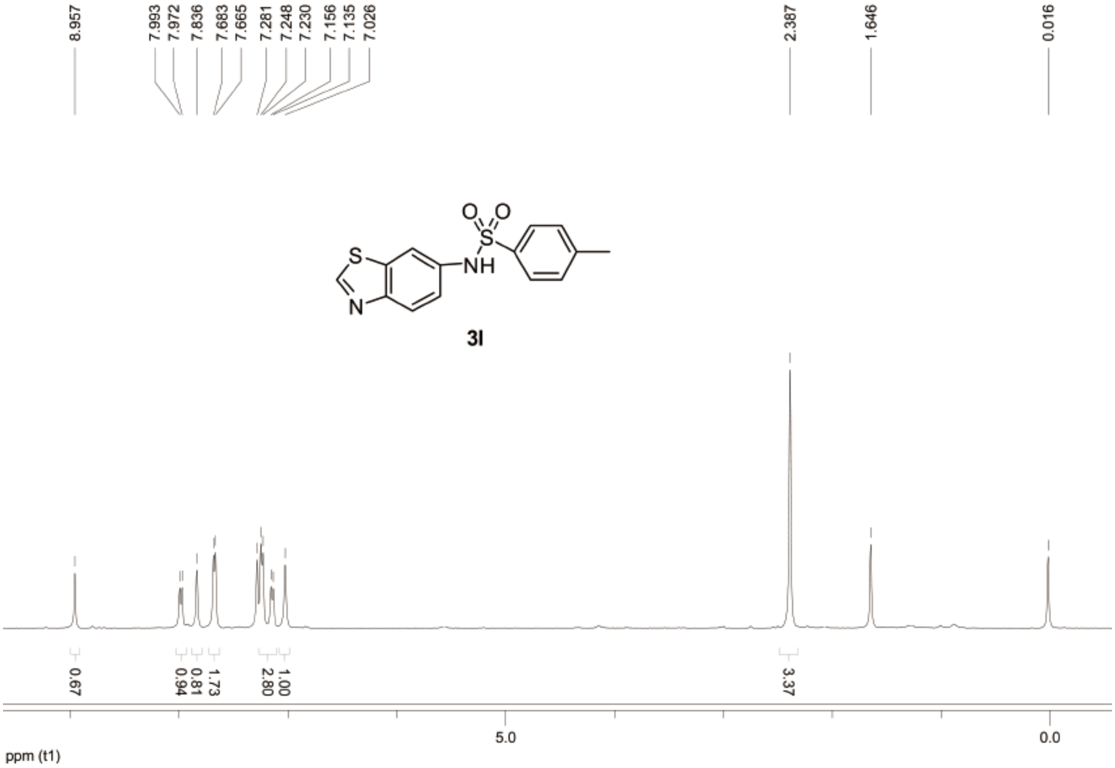
8.957
7.993
7.972
7.836
7.683
7.665
7.281
7.248
7.230
7.156
7.135
7.026
2.387
1.646
0.016
3l
0.67
0.94
0.81
1.73
2.80
1.00
3.37
5.0
0.0
ppm (t1)

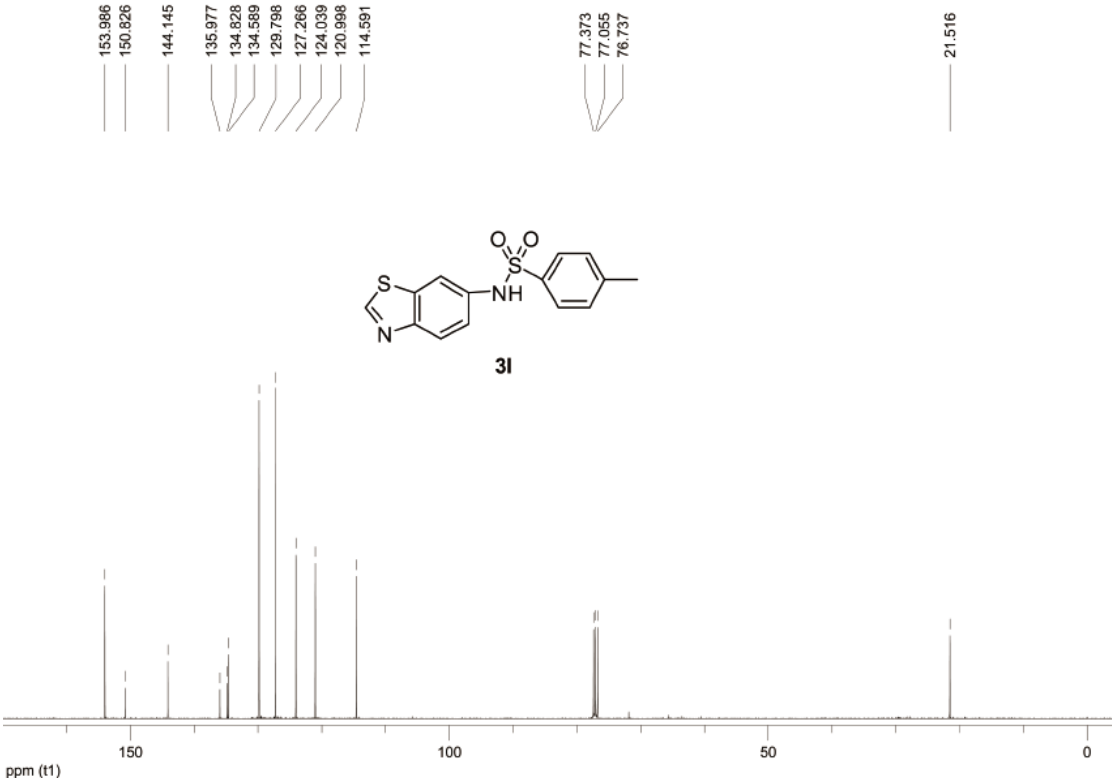
153.986
150.826
144.145
135.977
134.828
134.589
129.798
127.266
124.039
120.998
114.591
77.373
77.055
76.737
21.516
3l
150
100
50
0
ppm (t1)

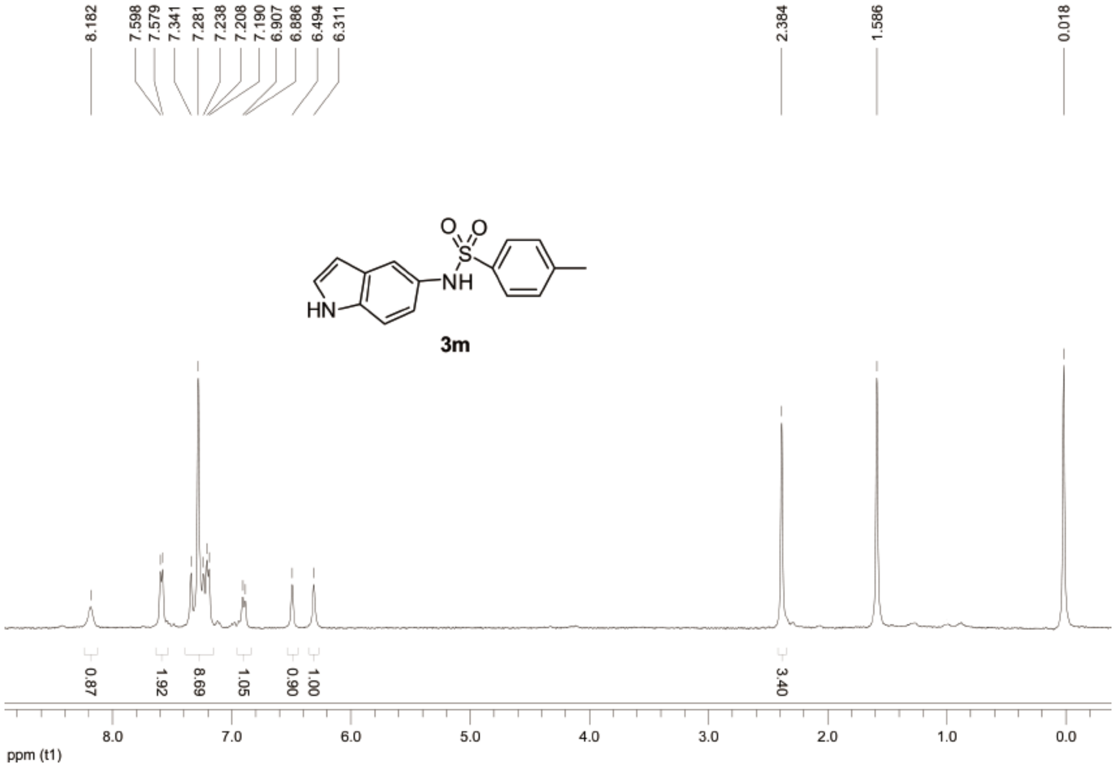
8.182
7.598
7.579
7.341
7.281
7.238
7.208
7.190
6.907
6.886
6.494
6.311
2.384
1.586
0.018
O
O
S
NH
HN
3m
0.87
1.92
8.69
1.05
0.90
1.00
3.40
8.0
7.0
6.0
5.0
4.0
3.0
2.0
1.0
0.0
ppm (t1)

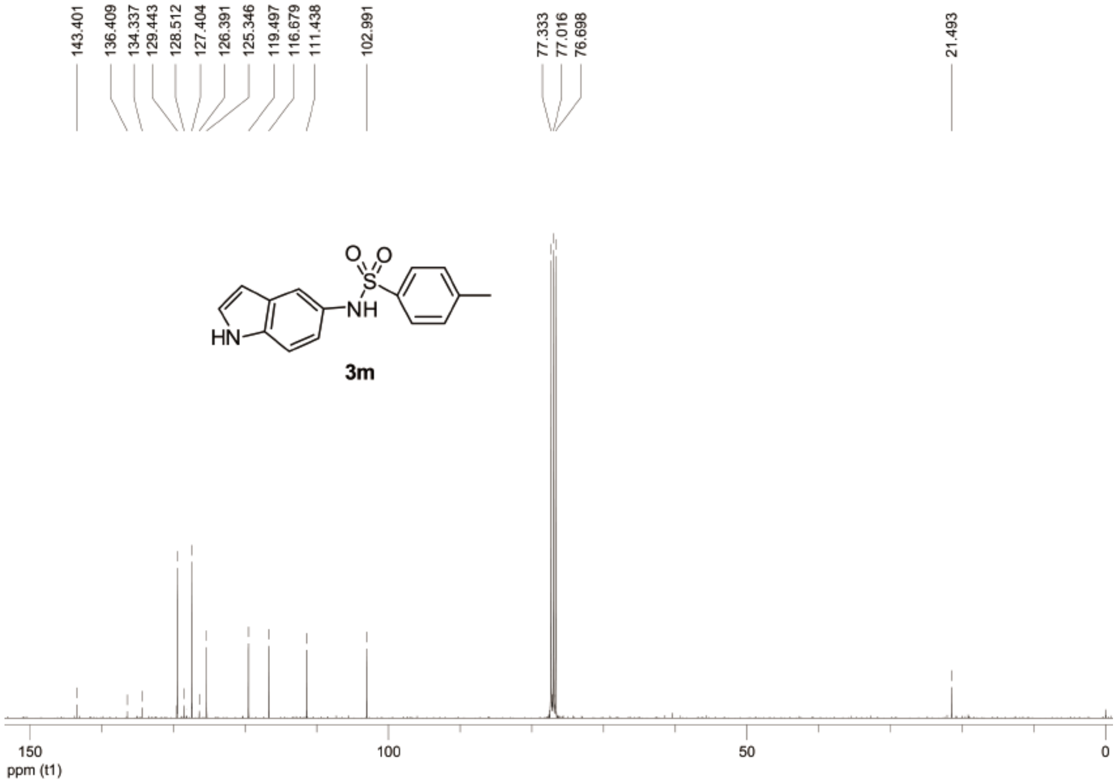
143.401
136.409
134.337
129.443
128.512
127.404
126.391
125.346
119.497
116.679
111.438
102.991
77.333
77.016
76.698
21.493
O
O
S
NH
HN
3m
150
100
50
0
ppm (t1)

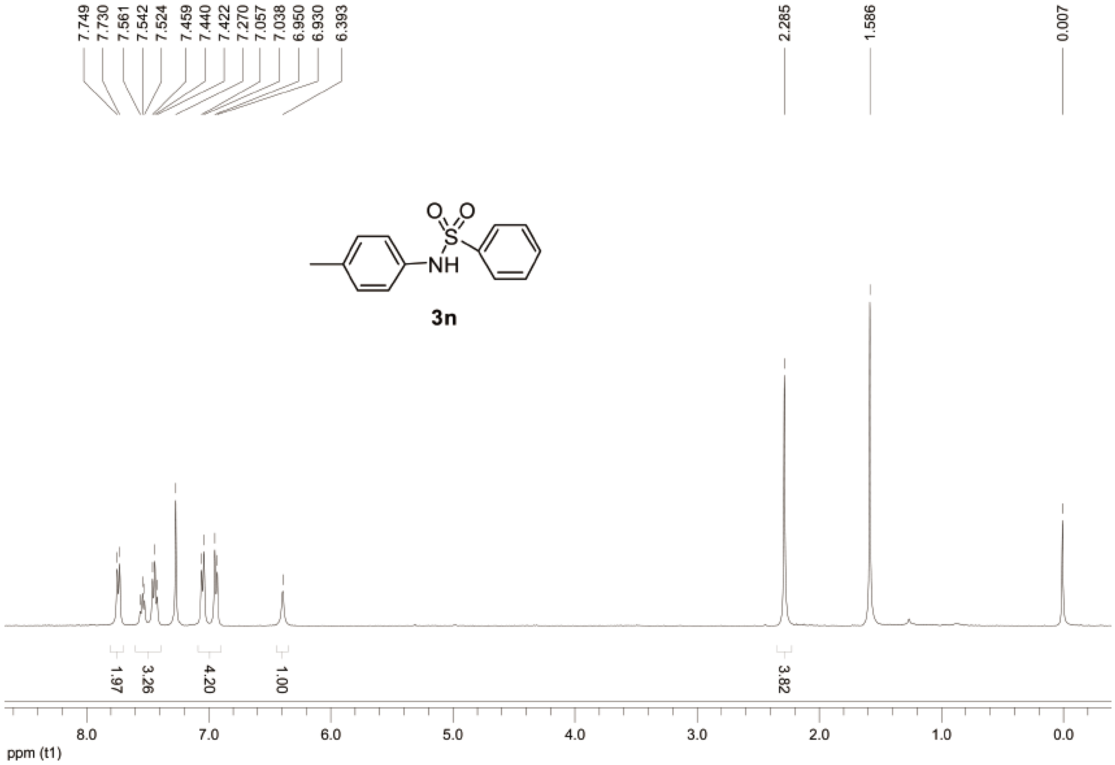
7.749
7.730
7.561
7.542
7.524
7.459
7.440
7.422
7.270
7.057
7.038
6.950
6.930
6.393
2.285
1.586
0.007
NH
S
O
O
3n
1.97
3.26
4.20
1.00
3.82
8.0
7.0
6.0
5.0
4.0
3.0
2.0
1.0
0.0
ppm (t1)

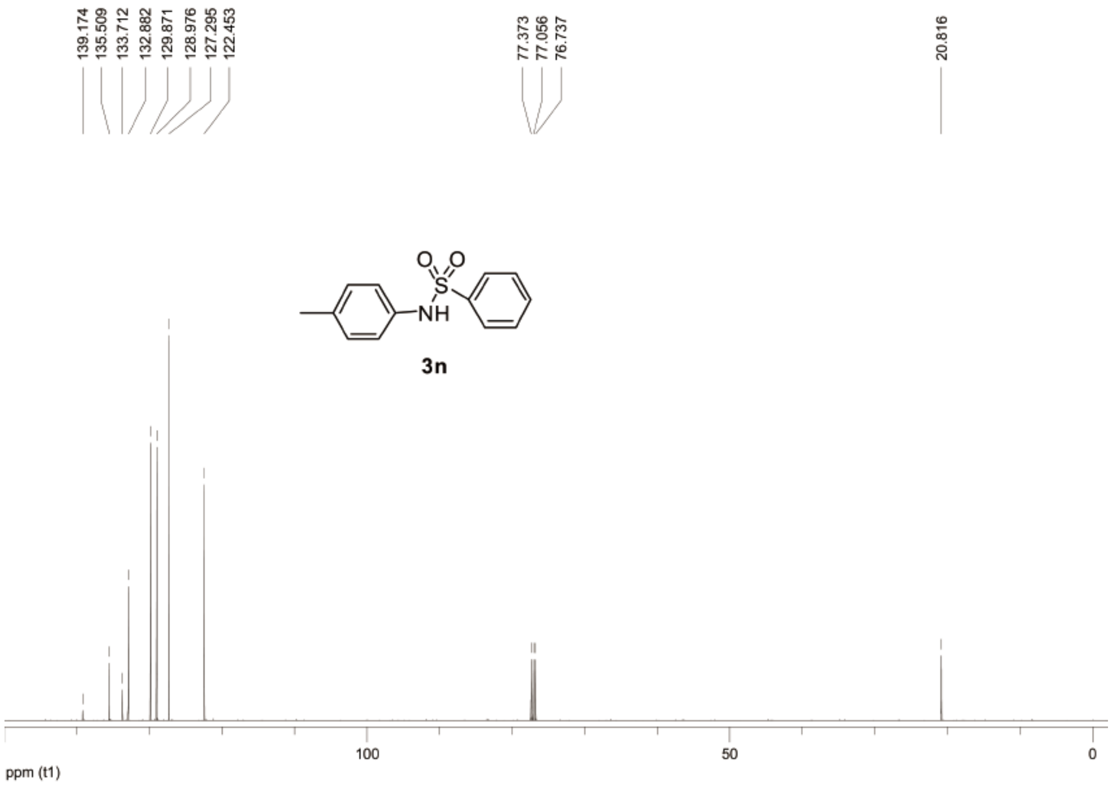
139.174
135.509
133.712
132.882
129.871
128.976
127.295
122.453
77.373
77.056
76.737
20.816
NH
S
O
O
3n
100
50
0
ppm (t1)

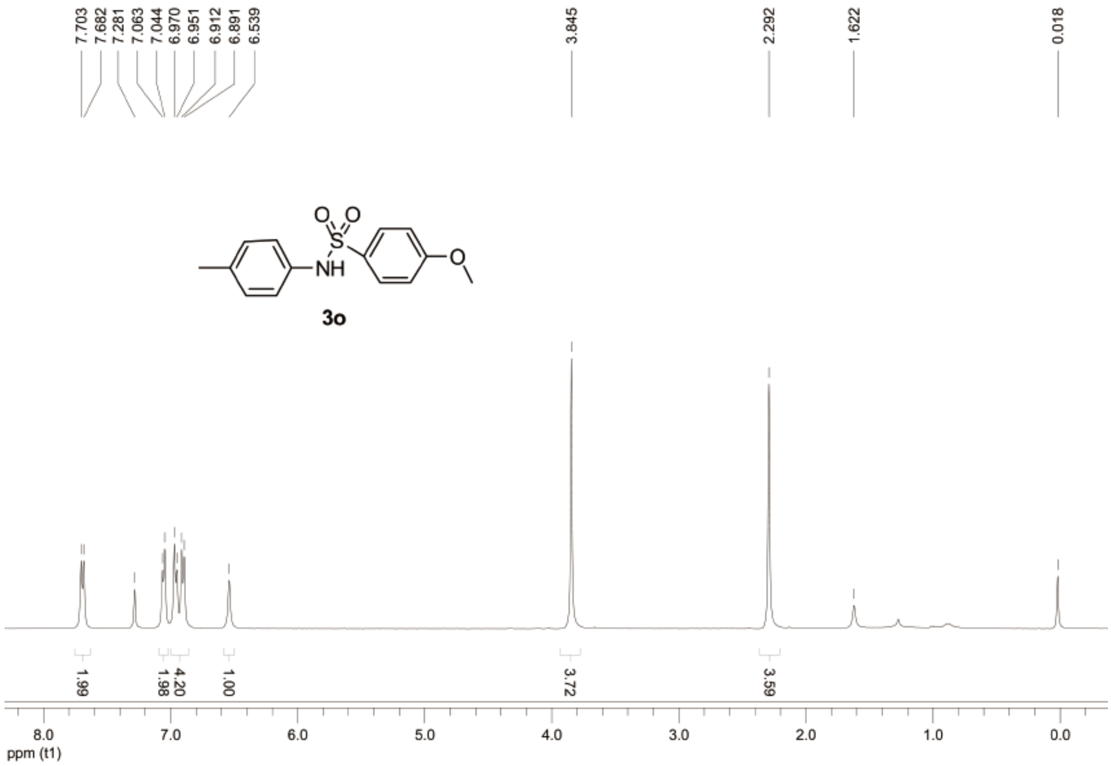
7.703
7.682
7.281
7.063
7.044
6.970
6.951
6.912
6.891
6.539
3.845
2.292
1.622
0.018
NH
O
S
O
3o
1.99
1.98
4.20
1.00
3.72
3.59
8.0
7.0
6.0
5.0
4.0
3.0
2.0
1.0
0.0
ppm (t1)

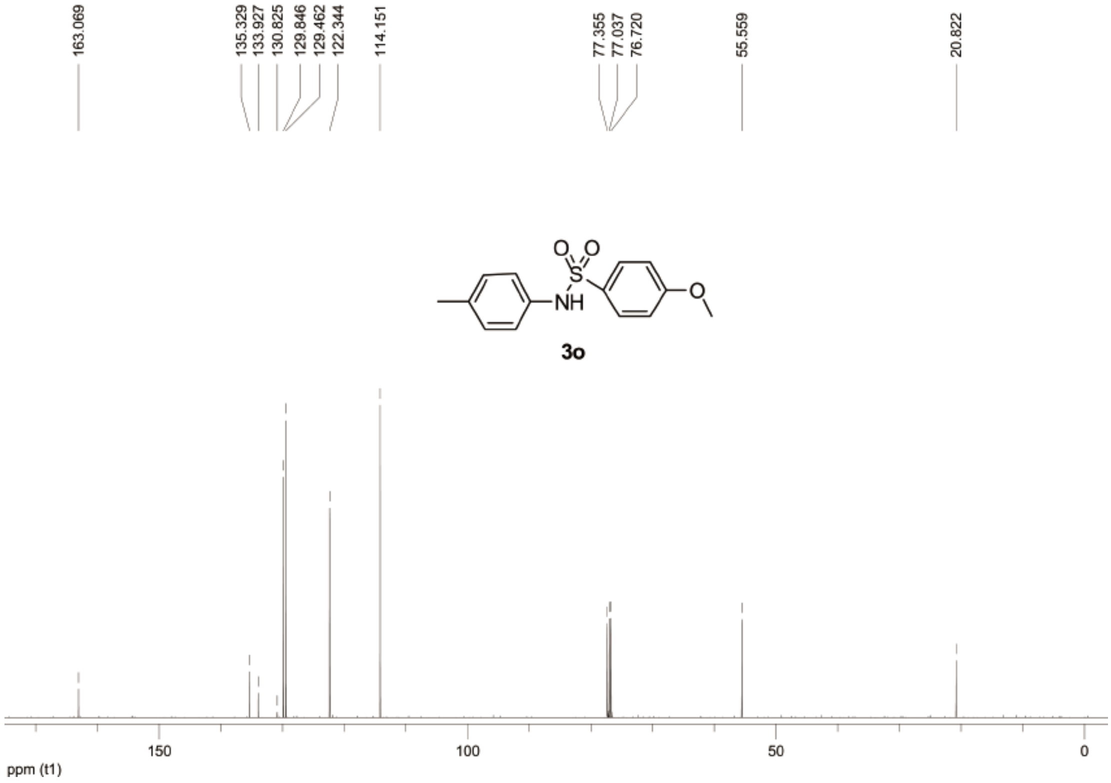
163.069
135.329
133.927
130.825
129.846
129.462
122.344
114.151
77.355
77.037
76.720
55.559
20.822
NH
O
S
O
3o
150
100
50
0
ppm (t1)

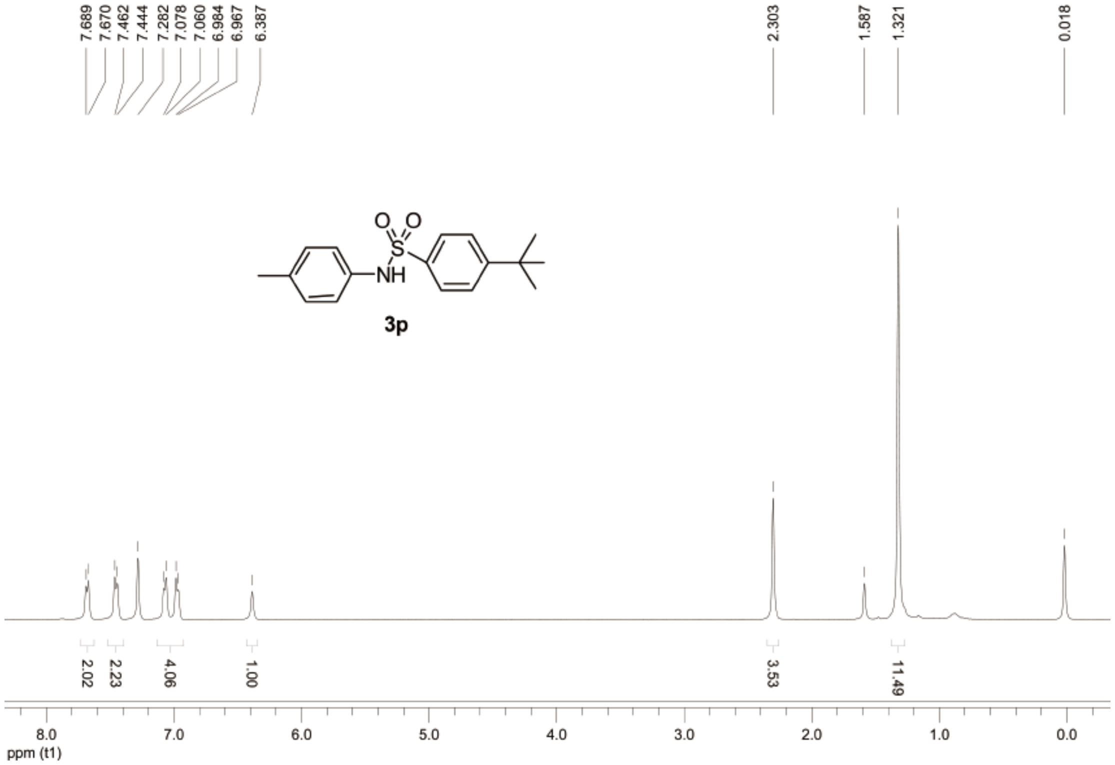
7.689
7.670
7.462
7.444
7.282
7.078
7.060
6.984
6.967
6.387
2.303
1.587
1.321
0.018
O
O
S
NH
3p
2.02
2.23
4.06
1.00
3.53
11.49
8.0
7.0
6.0
5.0
4.0
3.0
2.0
1.0
0.0
ppm (t1)

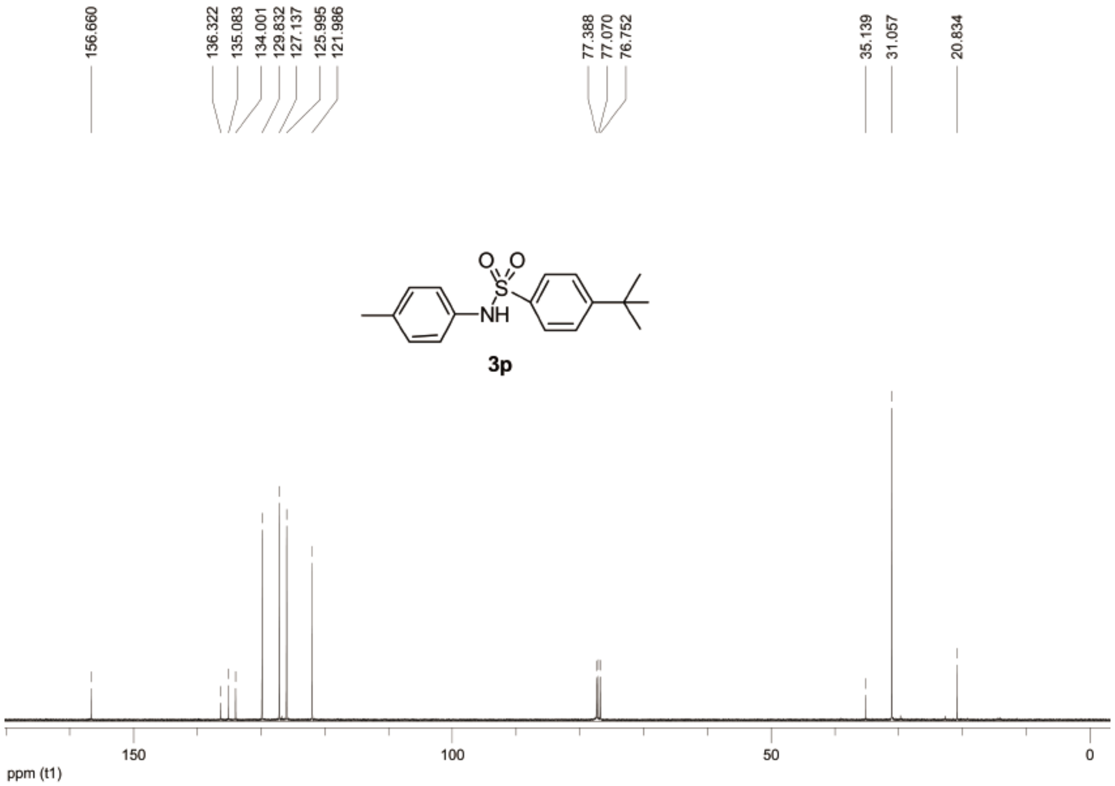
156.660
136.322
135.083
134.001
129.832
127.137
125.995
121.986
77.388
77.070
76.752
35.139
31.057
20.834
O
O
S
NH
3p
150
100
50
0
ppm (t1)

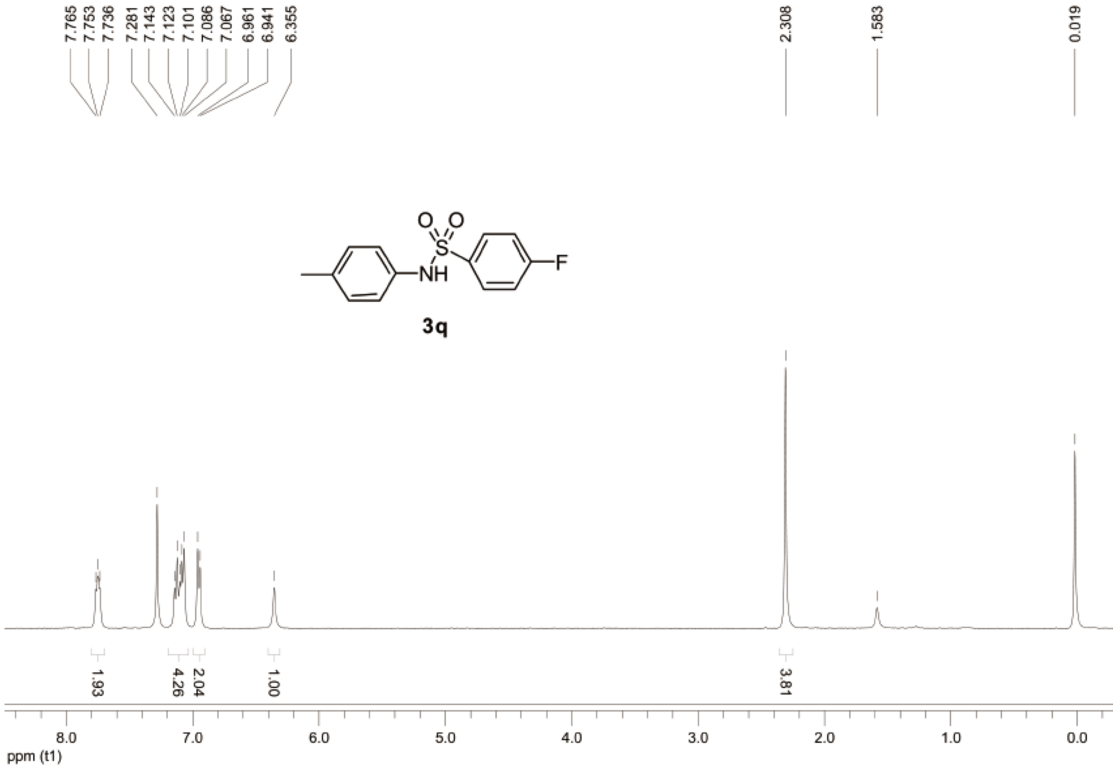
7.765
7.753
7.736
7.281
7.143
7.123
7.101
7.086
7.067
6.961
6.941
6.355
2.308
1.583
0.019
O
O
S
NH
F
3q
1.93
4.26
2.04
1.00
3.81
8.0
7.0
6.0
5.0
4.0
3.0
2.0
1.0
0.0
ppm (t1)

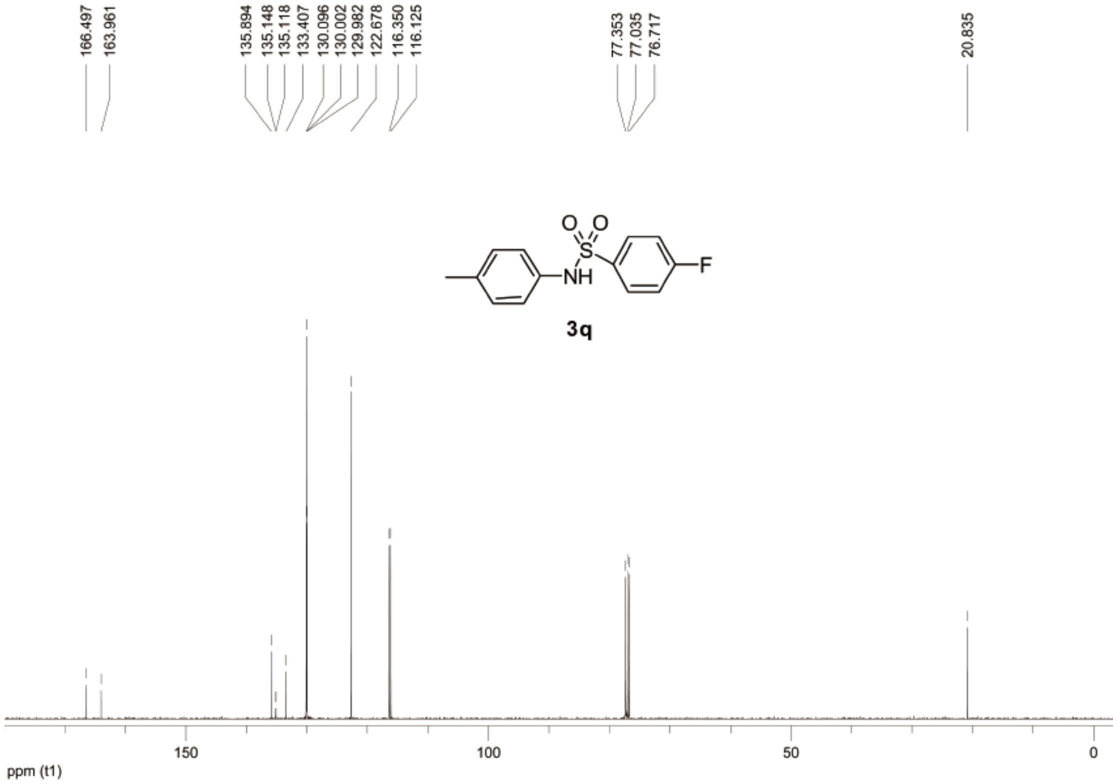
166.497
163.961
135.894
135.148
135.118
133.407
130.096
130.002
129.982
122.678
116.350
116.125
77.353
77.035
76.717
20.835
O
O
S
NH
F
3q
150
100
50
0
ppm (t1)

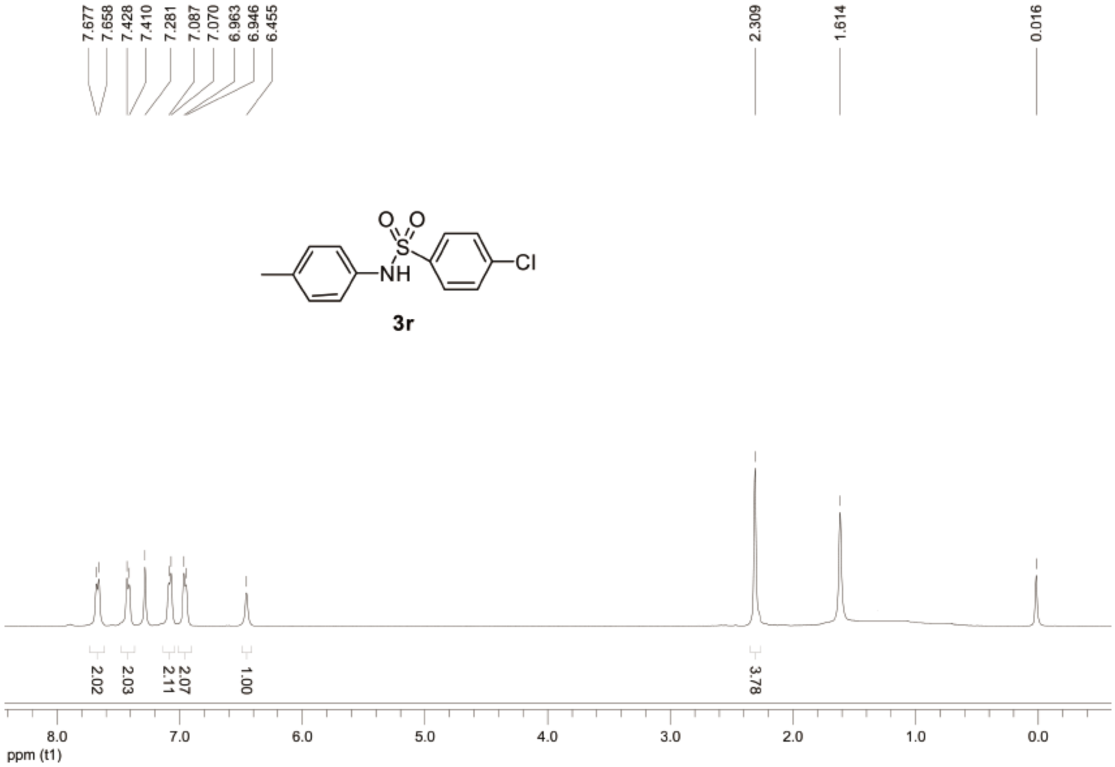
7.677
7.658
7.428
7.410
7.281
7.087
7.070
6.963
6.946
6.455
2.309
1.614
0.016
O O
S
NH
Cl
3r
2.02
2.03
2.11
2.07
1.00
3.78
8.0
7.0
6.0
5.0
4.0
3.0
2.0
1.0
0.0
ppm (t1)

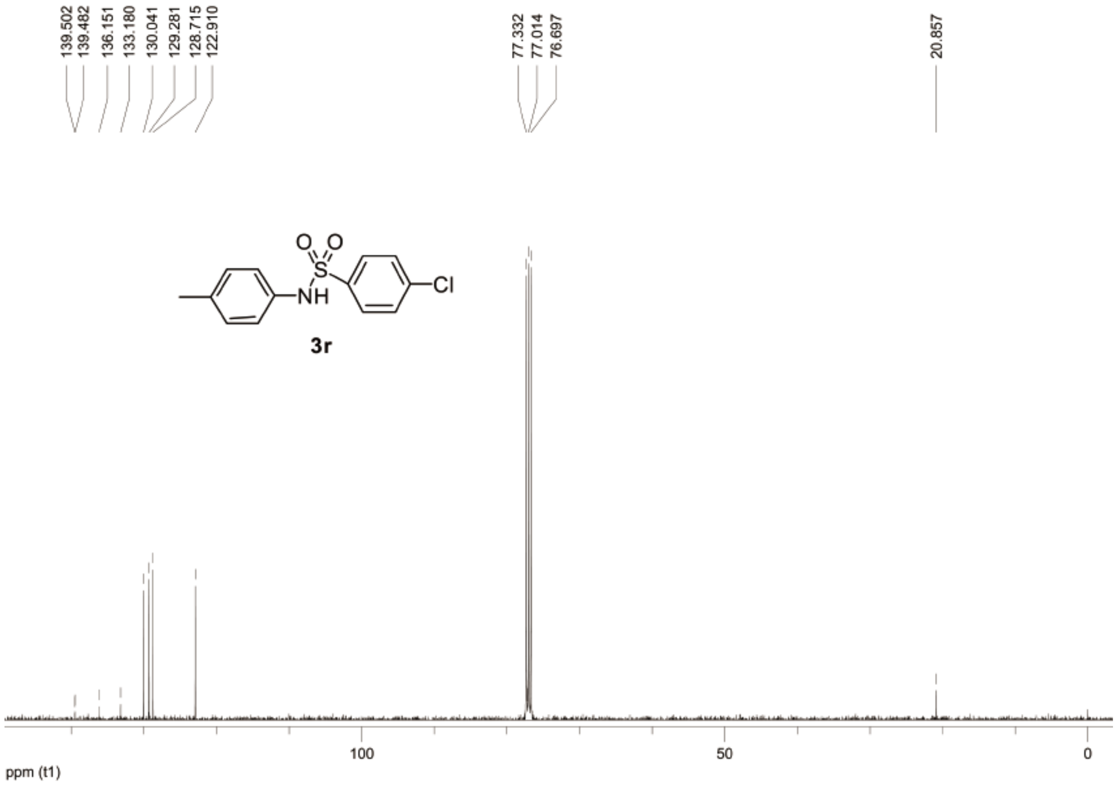
139.502
139.482
136.151
133.180
130.041
129.281
128.715
122.910
77.332
77.014
76.697
20.857
O O
S
NH
Cl
3r
100
50
0
ppm (t1)

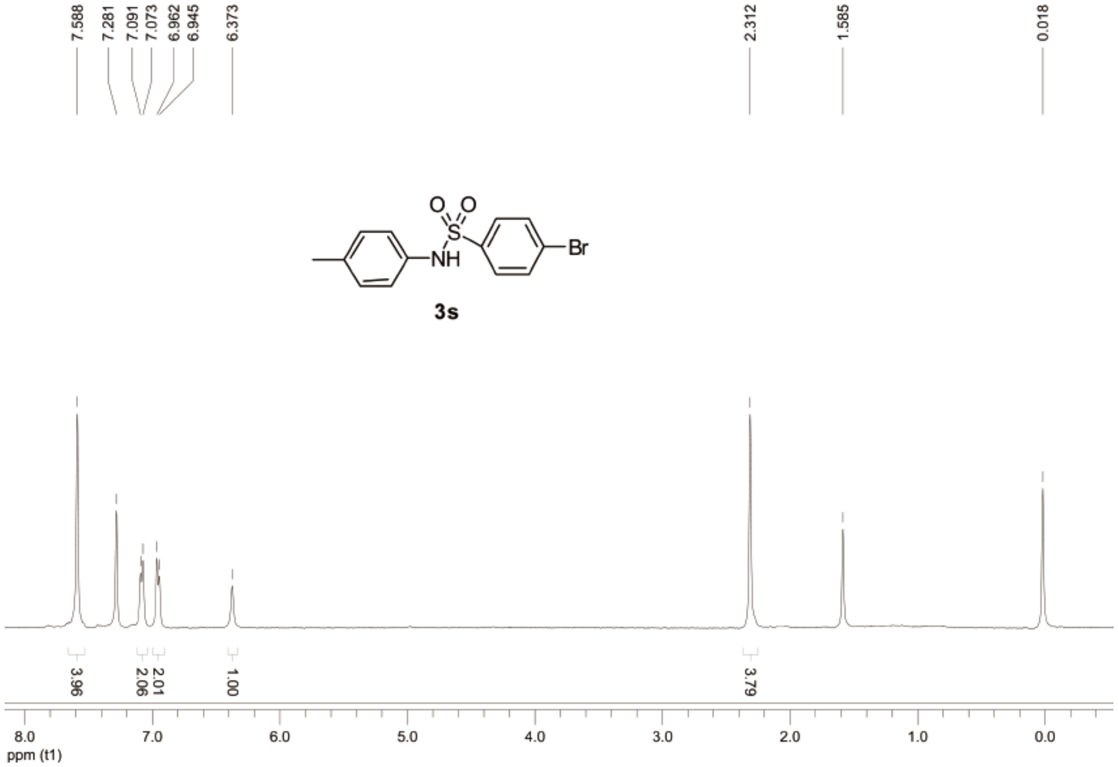
7.588
7.281
7.091
7.073
6.962
6.945
6.373
2.312
1.585
0.018
O
O
S
NH
Br
3s
3.96
2.06
2.01
1.00
3.79
8.0
7.0
6.0
5.0
4.0
3.0
2.0
1.0
0.0
ppm (t1)

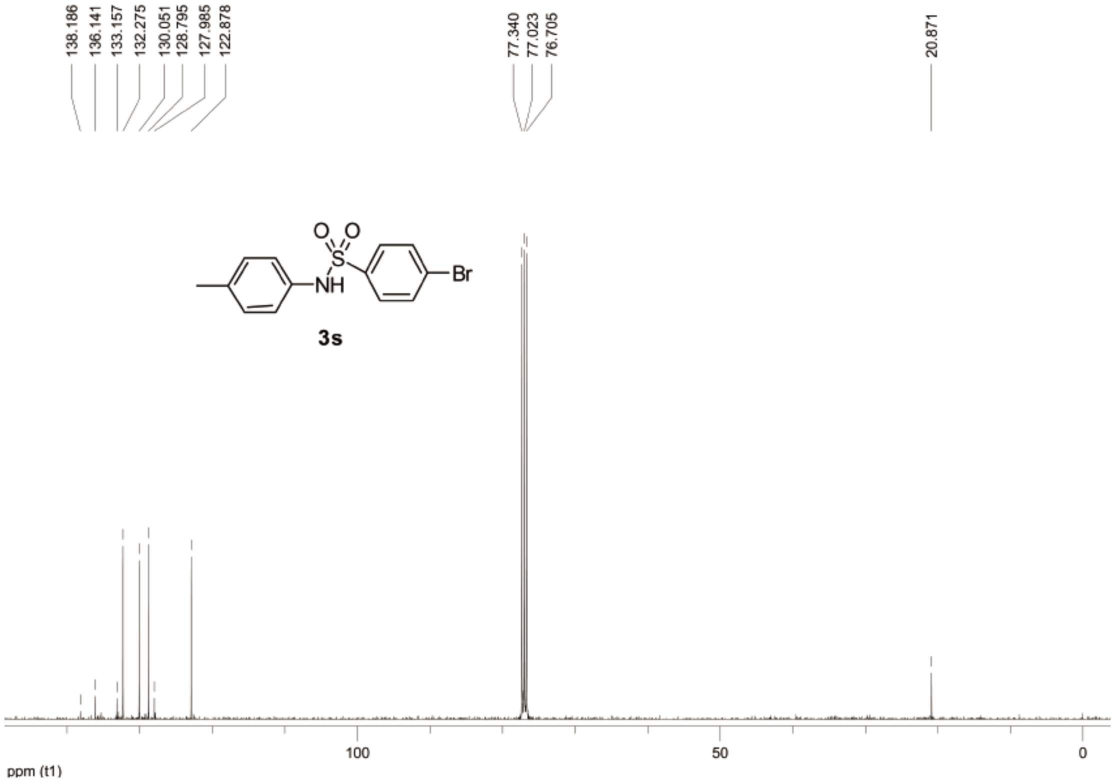
138.186
136.141
133.157
132.275
130.051
128.795
127.985
122.878
77.340
77.023
76.705
20.871
O
O
S
NH
Br
3s
100
50
0
ppm (t1)

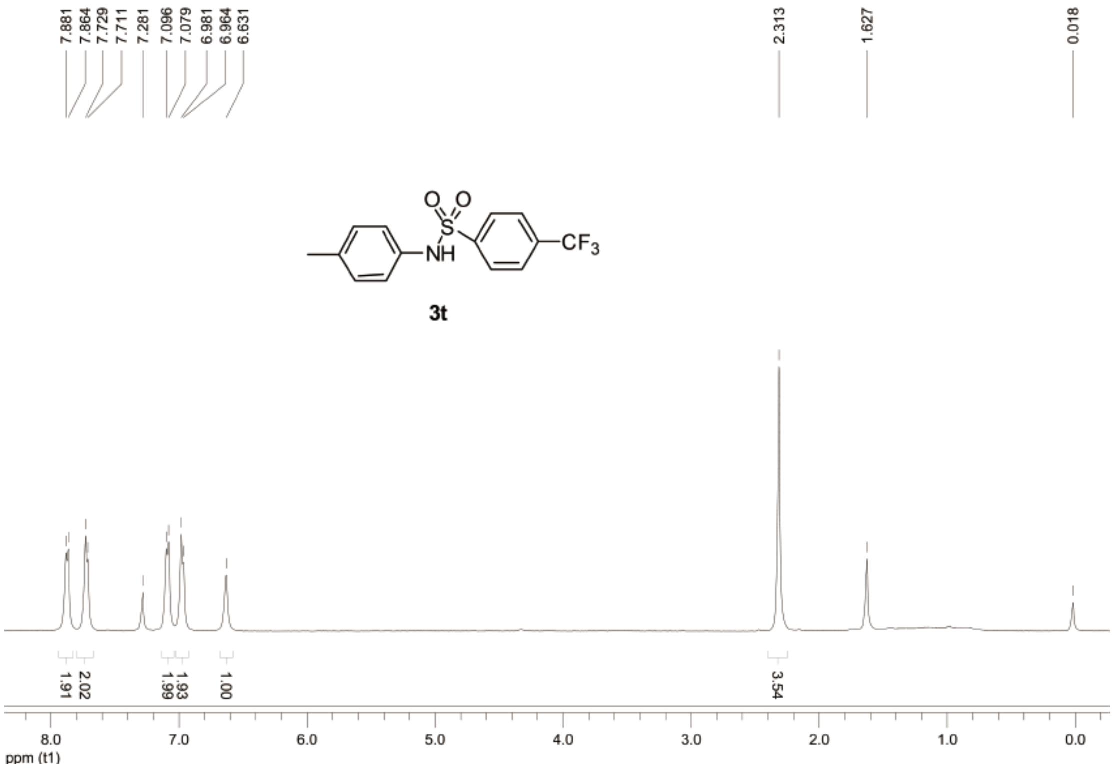
7.881
7.864
7.729
7.711
7.281
7.096
7.079
6.981
6.964
6.631
2.313
1.627
0.018
NH
CF3
3t
1.91
2.02
1.99
1.93
1.00
3.54
8.0
7.0
6.0
5.0
4.0
3.0
2.0
1.0
0.0
ppm (t1)

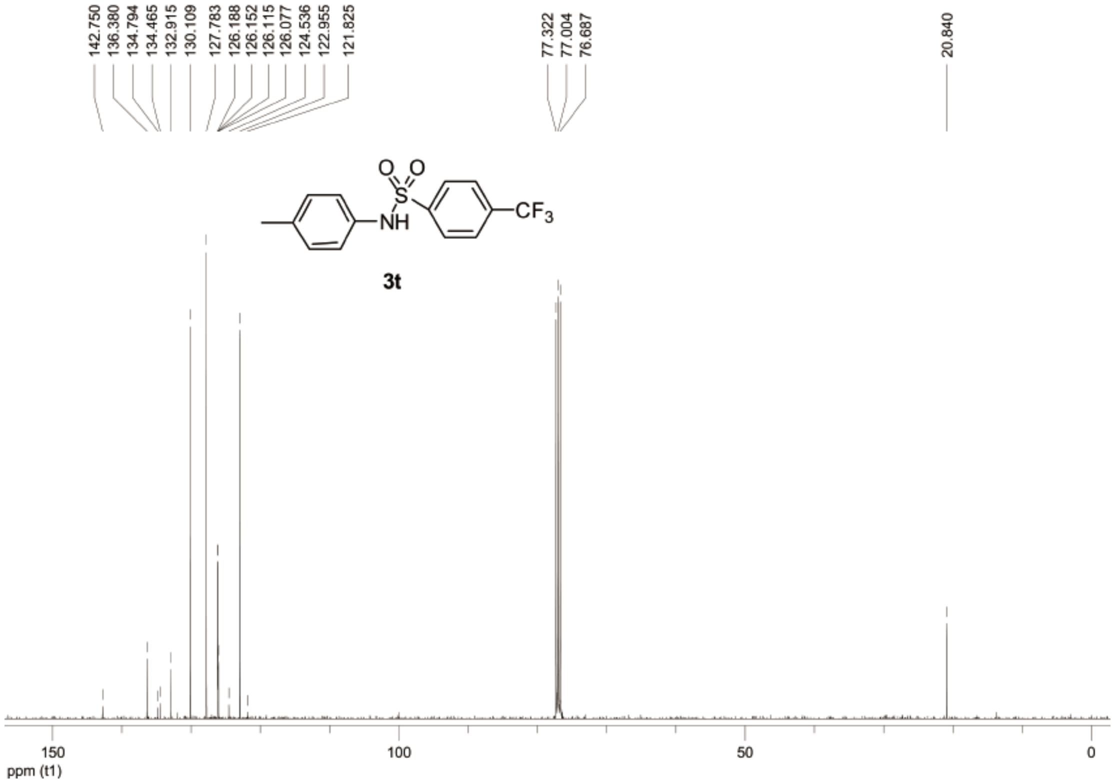
142.750
136.380
134.794
134.465
132.915
130.109
127.783
126.188
126.152
126.115
126.077
124.536
122.955
121.825
77.322
77.004
76.687
20.840
NH
CF3
3t
150
100
50
0
ppm (t1)

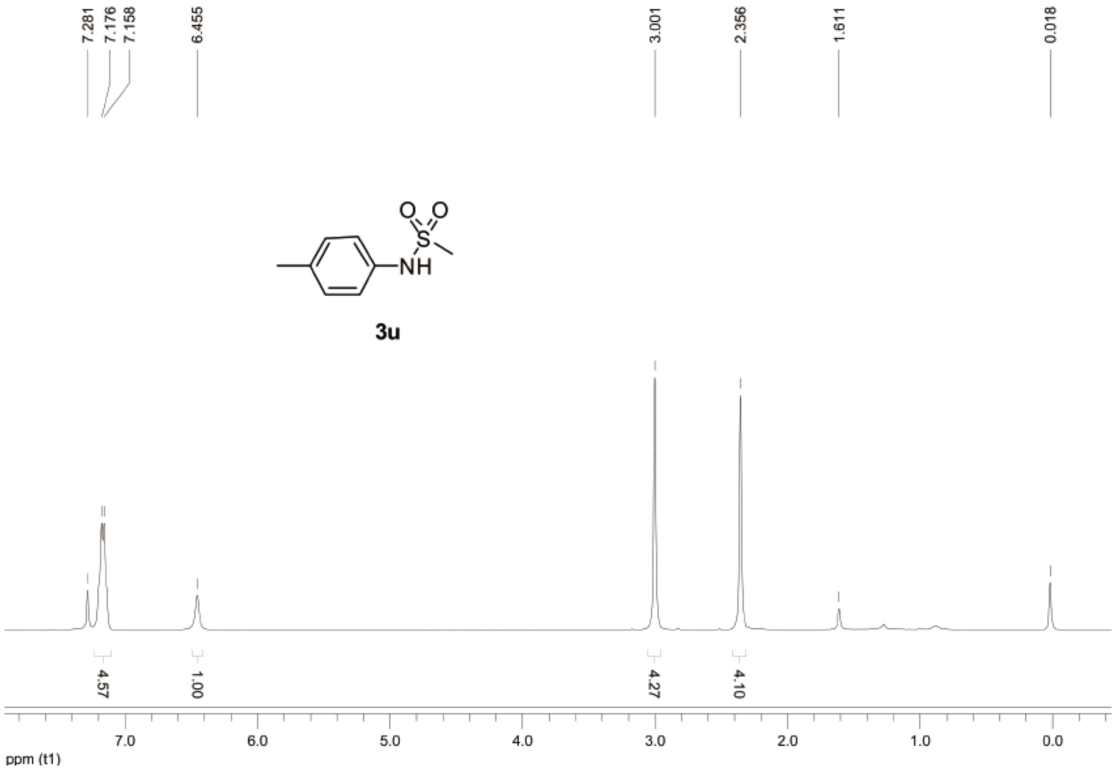
7.281
7.176
7.158
6.455
3.001
2.356
1.611
0.018
NH
3u
4.57
1.00
4.27
4.10
7.0
6.0
5.0
4.0
3.0
2.0
1.0
0.0
ppm (t1)

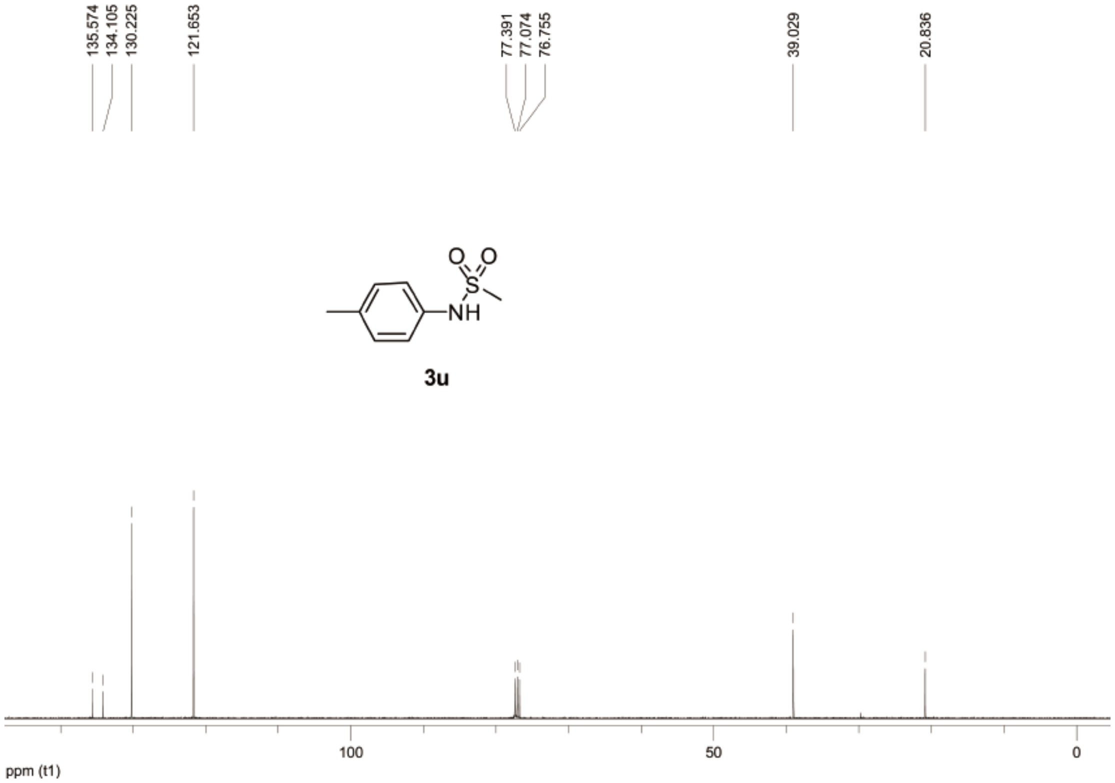
135.574
134.105
130.225
121.653
77.391
77.074
76.755
39.029
20.836
NH
3u
100
50
0
ppm (t1)
